# The related EIF4G3 and EIF4G4 initiation factors from *Leishmania*: dissimilar modes of action during translation revealed by a comparative proteomic approach

**DOI:** 10.1186/s13071-026-07297-1

**Published:** 2026-03-04

**Authors:** Stéphanny Sallomé Sousa Oliveira, Larissa Melo do Nascimento, Danielle Maria Nascimento Moura, Christian Robson de Souza Reis, Osvaldo Pompílio de Melo Neto

**Affiliations:** 1 Fundação Oswaldo Cruz, Fiocruz, Instituto Aggeu Magalhães, Recife, Pernambuco Brazil; 2 Universidade de Pernambuco, Recife, Pernambuco Brazil

**Keywords:** Protein synthesis, mRNA translation, eIF4F complex, eIF4G, RNA binding protein, Mass spectrometry

## Abstract

**Background:**

The start of eukaryotic translation requires recruitment of messenger RNA (mRNA) through the action of the eukaryotic initiation factor 4F (eIF4F) complex. eIF4F is formed by joining of the eIF4G and eIF4E subunits and generally also requires the eIF4A helicase. In *Leishmania infantum*, five eIF4Gs form multiple eIF4F-like complexes, with those based on the related EIF4G3 and EIF4G4 being active during translation, but with likely nonredundant roles that need to be better defined.

**Methods:**

To further investigate the roles of EIF4G3 and EIF4G4 in *Leishmania infantum*, we generated transgenic cell lines expressing each protein tagged with a C-terminal hemagglutinin (HA) epitope. Expression analyses were then carried out during different phases of promastigote growth, followed by gene knockout and complementation assays investigating the essentiality of the targeted eIF4Gs. The HA-tagged proteins were then used as baits in a large-scale investigation of potential protein partners, from different growth phases: early exponential, late exponential, and stationary.

**Results:**

EIF4G3 and EIF4G4 were expressed as multiple isoforms during promastigote growth, with EIF4G4 isoforms changing according to the growth phase. The two HA-tagged proteins were capable of replacing the corresponding native proteins after deletion of the endogenous genes. EIF4G3-HA and EIF4G4-HA were always found with their known eIF4E partners, respectively EIF4E4 and EIF4E3. EIF4G3-HA also more consistently coprecipitated with poly(A)-binding protein 1 (PABP1), RNA-binding protein 23 (RBP23), and EIF4AI, with EIF4G4-HA having greater association with PABP3 and the HEL67 helicase. A variable number of translation factors and ribosomal proteins were found with both baits, reflecting roles in translation. Our extensive analyses, investigating also proteins with possible moonlighting roles and uncharacterized polypeptides, not only revealed new proteins bound to both baits but also identified new specific partners for EIF4G3, and possibly EIF4G4, some of those being restricted to selected growth phases.

**Conclusions:**

Overall, new and more defined binding partners were observed for EIF4G3, with EIF4G4 having an increased coprecipitation with other translation initiation factors. Newly identified partners, for both eIF4Gs, might facilitate specific mRNA recognition or function regulating translation during growth. Further studies on some of those might reveal unique and conserved aspects of the *Leishmania* translation and might help define targets for novel translation inhibitors.

**Graphical Abstract:**

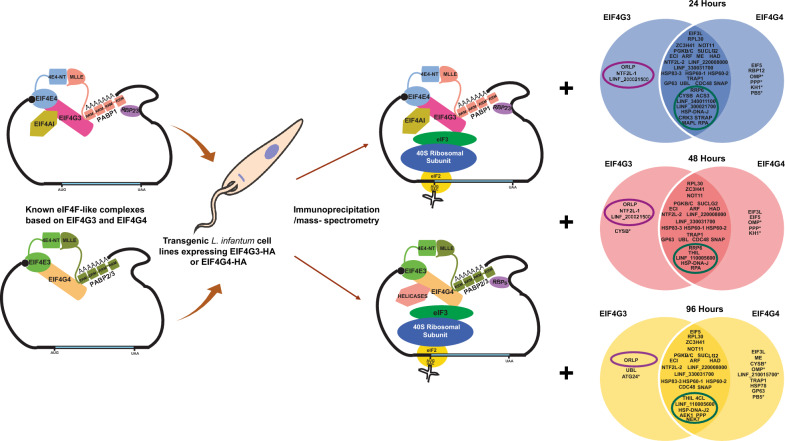

**Supplementary Information:**

The online version contains supplementary material available at 10.1186/s13071-026-07297-1.

## Background

Protein synthesis, or translation, is an essential process that, in eukaryotes, requires the actions of numerous polypeptides, RNA molecules, and the 40S and 60S ribosomal subunits. The initiation of translation is a critical stage, dependent on the action of many different eukaryotic initiation factors (eIFs) [1–3]. One of those, the eIF4F complex, binds to the capped 5′ end of the mRNA and, through a simultaneous interaction with the large multi-subunit eIF3 complex, facilitates the recruitment to the mRNA of the 40S ribosomal subunit, allowing translation to proceed. eIF4F is formed by the joining of the eIF4E and eIF4G subunits, with eIF4E binding to the 5′ cap while eIF4G mediates the interaction with eIF3 and other protein partners. A third eIF4F subunit in mammals, more loosely associated in yeast, is the RNA helicase eIF4A, an enzyme that removes interfering secondary structures on the mRNA 5′ UTR [4–7]. An important eIF4F partner is the poly(A) binding protein (PABP), with a prominent role in translation initiation through its binding to the 3′ end of the mRNA and its simultaneous interaction with eIF4G [8–11].

Multiple eIF4F-like complexes, consisting of different eIF4E and eIF4G subunits, have been shown to coexist in the same organism, with the best-known examples studied in mammals and plants [12–15]. A unique example of eIF4F diversity, however, has been found in trypanosomatid protozoans, best known by the genera *Leishmania* and *Trypanosoma,* and which include many species pathogenic to humans [16]. Various eIF4Es (six, EIF4E1 through EIF4E6) and eIF4Gs (five, EIF4G1 through EIF4G5) have been described from these unicellular parasites, forming multiple and distinct eIF4F-like complexes [17]. These are likely to exhibit relevant differences as well as similarities with the eIF4F mode of action in other eukaryotes.

Trypanosomatids diverged very early from major eukaryotic lineages and developed a unique set of molecular features, including polycistronic transcription and *trans*-splicing of all mRNAs [18]. Constitutive transcription of most protein-encoding mRNAs requires that the regulation of gene expression in these unicellular eukaryotes occurs mainly through posttranscriptional and posttranslational mechanisms. mRNA translation is therefore a critical target for regulating mechanisms required for proper control of gene expression [19]. These organisms then emerge as relevant models for the study of mechanisms required for eukaryotic translation and its regulation.

All five trypanosomatid eIF4Gs share a central, conserved, MIF4G/HEAT1 domain, but apart from EIF4G3 and EIF4G4, which share conserved segments within their N- and C-terminal regions, no other similarity in sequence is seen between different eIF4G homologs [17, 20–22]. These two eIF4Gs bind specifically to related eIF4E partners, EIF4G3 to EIF4E4 and EIF4G4 to EIF4E3, which share features not found in other eIF4Es, thus forming two related but distinct eIF4F-like complexes [21, 23–25]. Both EIF4G3 and EIF4G4 were also found to be able to bind to the trypanosomatid eIF4A homolog, EIF4AI, but with a possibly stronger interaction for the EIF4G3/EIF4AI pair [20, 21]. The interactions between these eIF4Gs and the three *Leishmania* PABP homologs have also been investigated, with *in vitro *results supporting a direct interaction between EIF4G3 and PABP1 only [26].

EIF4G3 and EIF4G4 are essential for cell viability in *Trypanosoma brucei*, and depletion of EIF4G3 drastically inhibits protein synthesis, while depletion of EIF4G4 does not cause evident changes in translation, despite leading to changes in cell morphology suggestive of interference with the cell cycle [21]. mRNA tethering assays performed in *T. brucei* further revealed that both eIF4Gs and their eIF4E partners can enhance expression of a reporter mRNA [27], with all four proteins found associated with polysomes in sucrose gradients [28]. In *Leishmania*, EIF4E4, EIF4G3, and EIF4E3, but not EIF4G4, were all found with polysomes [23, 25, 29]. These results are compatible with EIF4G3 and the associated complex having a direct role during translation initiation, and although they also implicate EIF4G4, its function is not as clearly defined.

Recent data from both *T. brucei* and *Leishmania* strongly suggest that different mRNA targets are associated with the EIF4E3/EIF4G4 and EIF4E4/EIF4G3 complexes [30, 31]. Previous studies have also investigated specific protein partners associated with both sets of EIF4E3/EIF4G4 and EIF4E4/EIF4G3 subunits in *Trypanosoma* species [31, 32], with the corresponding eIF4Es also having been investigated in *Leishmania* [25, 33]. These studies helped define a limited set of additional protein partners mostly found with the EIF4E4/EIF4G3 complex (EIF4AI, PABP1, and the RNA-binding protein named RBP23). The known partners, however, are unlikely on their own to define binding to different mRNA targets or mediate specific regulatory events associated with changes in translation levels. Therefore, aiming to investigate further the questions raised regarding the functional differences between EIF4G3 and EIF4G4, here we opted to use transgenic *Leishmania infantum* cell lines ectopically expressing each of these two eIF4Gs tagged with a C-terminal HA epitope. The two HA-tagged proteins were expressed with a pattern of multiple isoforms, indicative of posttranslational modifications, and were also able to complement the deletion of both copies of the corresponding endogenous genes, found to be essential for the promastigote stage of the *Leishmania* lifecycle. To better define new proteins that might be functionally relevant for the associated complexes in *Leishmania*, either as direct partners or regulators, we next used a comparative approach to extensively investigate proteins coprecipitated with EIF4G3-HA or EIF4G4-HA. This approach also aimed to identify differences in coprecipitated proteins between different phases of the promastigote growth, and which represent contrasting translation levels, high and low. Our results reinforce roles for both EIF4G3 and EIF4G4, as well as their eIF4E partners, during translation initiation, while revealing a large number of known and new potential partners found with either or both eIF4Gs. Overall, the data are consistent with EIF4G3 having a more defined set of binding partners, including proteins not previously identified, which might be required for the recruitment of specific mRNA targets and might mediate regulatory events. In contrast, EIF4G4 does not seem to have such a specific set of defined partners, apart from EIF4E3, but might have a greater interaction with known translation initiation factors. Several potential new partners, including enzymes with possible moonlighting functions and uncharacterized polypeptides, were also identified for either or both complexes, with possible roles during mRNA binding and/or translation which will need further characterization. Multiple proteins were also found with a selective association with the targeted complexes only at specific growth phases, raising the hypothesis that they might be associated with mechanisms regulating translation during *Leishmania* growth, although these also remain to be further validated.

## Methods

### Cloning procedures

Gene sequences encoding *Leishmania infantum* (strain JPCM5) EIF4G3 (LINF_160022100) and EIF4G4 (LINF_360070500) were retrieved from TriTrypDB (https://tritrypdb.org). Gene amplifications were performed from *L. infantum* genomic DNA, as previously described [34, 35], using primers flanked by *BamH*I (5′ end) and *Hind*III (3′ end) restriction sites and the HA-coding sequence added to the 3′ primers immediately before a stop codon and the *Hind*III site (listed in Additional File 1: Supplementary Table 1). The amplified fragments were cloned into the pGEM-T Easy cloning vector (Promega), sequenced, and subcloned into the *BamH*I/*Hind*III sites of the pSP-BT1-Y-Neo-alpha expression vector [36]. The cassettes for the gene deletions were obtained through polymerase chain reaction (PCR) fusion reactions, also as previously described [34], using specific primers for the amplification of 5′ and 3′ regions flanking the target genes to be deleted, as well as sequences encoding the hygromycin and puromycin resistance markers (all also included in Additional File 1: Supplementary Table 1). These cassettes were then cloned into pGEM-T Easy vector (Promega) prior to their use during the gene deletion experiments.

### *Leishmania infantum* growth

The experiments were carried out with *Leishmania infantum* MHOM/MA/67/ITMAP-263 promastigotes, cultured at 26 °C in Schneider medium pH 7.2, supplemented with 10% heat-inactivated fetal bovine serum (FBS) and 0.25% v/v hemin solution. For the growth curves of nontransfected or transgenic cell lines, the cultures were kept until reaching the stationary phase, diluted in fresh medium to an initial concentration of 1.0 × 10^6^ cell/mL, and counted in a Neubauer chamber for the following five days. Total cell extracts were prepared daily to a final concentration of 2 × 10^5^ cells/µL to be analyzed by Western blots.

### Generation of *Leishmania infantum* transgenic cell lines

To generate the cell lines expressing the HA-tagged proteins, constructs in the pSP-BT1-Y-Neo-alpha vector (5–10 µg) were transfected into 2.0 × 10^8^ promastigotes by electroporation, as previously described [30], followed by selection of transfectants with G418 (80 µg/mL). To generate the knockout cell lines, the deletion cassettes cloned into pGEM-T easy plasmid were recovered through digestion with *Not*I (cassetes with the hygromycin resistance marker or hygR) and *EcoR*I (puromycin resistance marker or puroR) restriction enzymes followed by gel purification. The hygR fragments were first transfected into *L. infantum* promastigote cells, with single knockout (SKO) clones selected on Schneider-agar plates supplemented with hygromycin (80 µg/mL). For the generation of double knockout (DKO) cell lines for both EIF4G3 and EIF4G4, we attempted to replace the second gene copy by performing a second round of transfection using the SKO cell lines with the puroR cassette, followed by selection with hygromycin and puromycin (70 µg/mL).

To validate the deletion assays and also to check the functionality of the HA-tagged proteins, complementation experiments were then performed by transfecting the SKO cell lines with the corresponding wild-type genes encoded in the pSP-derived plasmids, followed by selection with hygromycin as well as G418, and subsequent confirmation of the expression of the HA-tagged proteins. Another round of transfection was then performed in the selected transfectants, now using the puromycin cassette, followed by selection in the presence of the three antibiotics, hygromycin, neomycin, and puromycin. No attempts were made to recover clones from the transfections with the HA-tagged proteins or the puromycin markers, but all double knockout and complementation experiments were performed at least twice with a minimum of two distinct SKO clones. To confirm the integration events, the recovered cells were then used for DNA extraction and PCR amplification of selected fragments corresponding to the native genes and/or selectable markers. These were carried out with primers also listed in Additional File 1: Supplementary Table 1 using the GoTaq^®^ G2 Flexi DNA polymerase (Promega).

### Western blots

To confirm the expression of selected proteins in whole protein extracts from the different cell lines, these were subjected to standard Western blot procedures. For the analyses of the endogenous proteins, previously described rabbit polyclonal antibodies raised against *Leishmania* EIF4G3, EIF4G4, and EIF4AI [20, 37] were used, at 1:500 dilution, followed by incubation with anti-rabbit immunoglobulin (IgG) conjugated to horseradish peroxidase (HRP; Jackson ImmunoResearch, JACK-115035003), diluted 1:5000. For the HA-tagged proteins, the membranes were incubated with anti-HA monoclonal antibody (Abcam^®^, ab18181) at dilution of 1:5000 and anti-mouse IgG-HRP diluted 1:3000, as secondary antibody. To validate the immunoprecipitation experiments, equivalent Western blots were also performed, assessing the immunoprecipitated samples with the anti-HA monoclonal antibody, followed by testing the same samples with rabbit antibodies directed against the *Leishmania* EIF4E3, EIF4E4, PABP1, and PABP3, as previously described [20, 26, 37]. All the described results are derived from a minimum of two experiments with uncropped images from the results shown, as well as from independent experiments, found in Additional File 2: Supplementary Figs. 1–4.

### Whole-cell lysate preparation and immunoprecipitation assays

*L. infantum* promastigotes from polyclonal transgenic lineages expressing the EIF4G3-HA or EIF4G4-HA were cultured for the three selected time points (24 h, 48 h, and 96 h), followed by harvesting of approximately 2 × 10^8^ cells. These were then washed once with phosphate-buffered saline (PBS) and resuspended in 500 µL of lysis buffer (20 mM 4-(2-hydroxyethyl)-1-piperazineethanesulfonic acid (HEPES)–KOH pH 7.4, 75 mM KCH_3_CO_2_, 4 mM Mg (CH_3_COO)_2_, 2 mM Dithiothreitol (DTT)) supplemented with 10% protease inhibitor (ethylenediaminetetraacetic acid (EDTA)-free EASYpack, from Roche). Cell lysis was carried out by cavitation, as described previously [30], followed by centrifugation for 10 min at ~ 17,000*g*, 4 °C. The supernatant (lysate), corresponding to the soluble fraction, was aliquoted and used for the immunoprecipitation assays. For all three time points, cultures of nontransfected cells were also set up and used to prepare equivalent cell lysates/soluble fractions to be used as negative controls. For the immunoprecipitation experiments, the cytoplasmic fractions were first incubated for 30 min at 4 °C with anti-HA magnetic beads (Pierce™). After incubation, the beads were harvested, washed three times with PBS, and resuspended in sodium dodecyl sulfate (SDS)-polyacrylamide gel electrophoresis (PAGE) sample buffer. The experiments were carried out using three replicates, for each of the time points for each of the tagged eIF4G baits and the corresponding negative control, with the single exception being the 24 h time point for the control samples, which used only two replicates.

### Mass spectrometry analysis

For identification of coprecipitated proteins, the immunoprecipitated samples were loaded into 12.5% SDS-PAGE gels, followed by electrophoresis only until the samples entered the resolving gel. After brief staining with Coomassie Blue R-250, the gel slices containing the stained bands were excised and submitted to in-gel tryptic digestion, followed by analysis of the eluted peptides through liquid nanochromatography (Liquid chromatography–tandem mass spectrometry (LC–MS/MS)) using a LTQ Orbitrap XL ETD mass spectrometer (ThermoScientific). The identification of peptides and proteins was carried out as previously described [30] on the basis of the *L. infantum* protein sequence database (strain JPCM5, version from 13 July 2018, available from the TriTrypDB database), with accession numbers defined according to TritrypDB, but with the protein nomenclature and annotation based mainly on orthologs previously described in *Trypanosoma brucei* (strain TREU927), while the suggested cellular localization data were derived from the TrypTag (http://tryptag.org) [38]. The abbreviations for the different proteins were mostly based on those found in the literature or, when not available or unknown, defined arbitrarily. Consolidated lists of all coprecipitated proteins, represented by one peptide in at least two of the three replicates for either of the tagged baits, on any of the three time points are included in Supplementary Table 2.

Two different approaches were used to evaluate the mass spectrometry data. First, the raw mass-spectrometry data were analyzed using Perseus software, version 2.1.3.0 [39]. Label-free quantification (LFQ) intensities were transformed by the software to a log_2_ scale, and missing values were imputed on the basis of a normal distribution centered around the detection limit of the mass spectrometer. Perseus was used for principal component analysis (PCA) of the data as well as for the statistical analysis, performed using Student’s *t*-test, comparing protein intensities between the tagged eIF4G baits with the corresponding control samples for each time point and considering the various biological replicates. The results were visualized through volcano plots, where the *x*-axis represents the differences in log_2_ intensities between the two baits and the *y*-axis displays the −log(*P*) values, indicating the statistical significance of the observed differences. The significance threshold was defined on the basis of a false discovery rate (FDR) of 0.05 and an *s*_0_ parameter of 0.1.

A second, independent analysis was also performed using the normalized intensity values derived from every coprecipitated protein found with each of the various replicates, time points and cell lines (26 samples in total). To normalize the data, and for each replicate, we first determined the sum of the intensities for all proteins coprecipitated within that replicate. The highest sum among the replicates was then defined and used to calculate ratios for the sums derived from each of the other replicates. These ratios, the normalization factors, were then multiplied by the individual intensities for the various proteins found within the corresponding replicates. Consolidated lists of all coprecipitated proteins, represented by one peptide in at least two of the three replicates for either of the tagged baits, on any of the three time points, were then generated (Additional File 3: Supplementary Table 2). As previously described [30, 31], average values from the three replicates from any of the time points, for either of the HA-tagged baits or the control, were then determined and used to calculate the intensity enrichment ratios for the two baits in comparison with the control samples. Lists of all proteins with enrichment ratios greater than 1.5-fold (represented by at least one peptide in two replicates from either of the tagged eIF4Gs from any of the time points) or 4-fold (represented by at least two peptides in two of the replicates from either of the tagged eIF4Gs from any of the time points) were also generated and used for further comparisons, as described in subsequent sections. In these lists, all coprecipitated proteins are colored according to different functional categories, such as “translation factors”, “metabolic enzymes”, “RNA binding proteins”, “uncharacterized proteins”, and so on, with subcategories also defined for “metabolic enzymes” and “uncharacterized proteins.”

## Results

### Expression analysis of native and HA-tagged EIF4G3 and EIF4G4

**Fig. 1:**
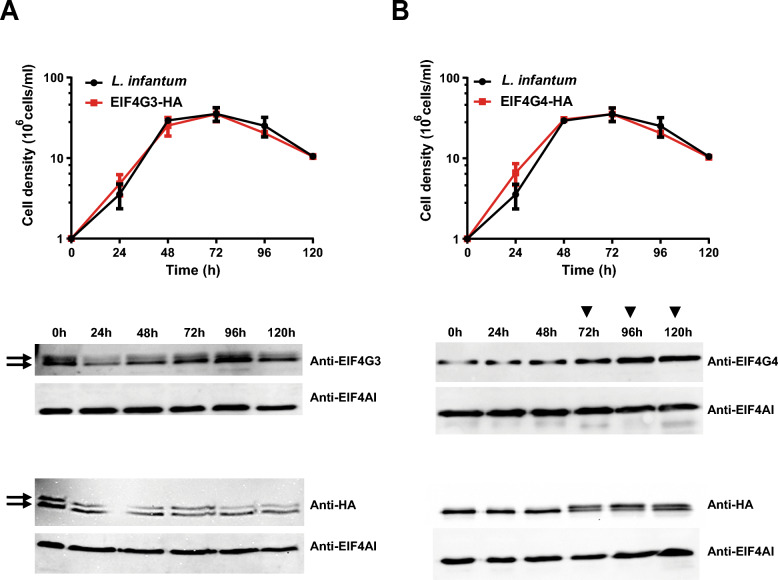
Expression analyses of the *Leishmania infantum* EIF4G3 and EIF4G4 during different growth phases of the promastigote life stage. Growth curves of wild-type *L. infantum* and transgenic cell lines expressing the HA- tagged EIF4G3 (**A**) or EIF4G4 (**B**) were set up using stationary phase cells diluted into fresh medium. Cell growth was monitored at 24-h intervals, when aliquots were also taken for Western blot analyses of whole-cell extracts. The graphs on the top left represent the cell growth, with the images from the Western blot analyses shown in the bottom. Detection of the native proteins in wild-type promastigotes was carried out using rabbit polyclonal anti-EIF4G3 and anti-EIF4G4 antibodies. The HA-tagged proteins, from the transgenic cell lines, were detected with a monoclonal anti-HA antibody. The native EIF4AI, probed with a polyclonal rabbit antiserum, was used as a loading control. In the Western blot images, the arrows highlight the EIF4G3 isoforms, with the ▼ symbol representing the time points at which the second EIF4G4 isoform is present

Both EIF4G3 and EIF4G4 are expressed in the two major life stages of the *T. brucei* lifecycle as well as in *Leishmania* promastigotes, generally represented by two or more isoforms, indicative of being targeted by posttranslational modifications [21, 25, 33, 37]. Different isoforms are also seen for their eIF4E partners in *Leishmania*, but these vary according to growth phase and/or stress [25, 29, 33, 34, 37]. To better define whether similar variations can also be seen for EIF4G3 or EIF4G4, we started our comparative analysis by evaluating their expression during different growth phases of *L. infantum* promastigotes. Western blots were then set up, first using antibodies directed against the native proteins to assess their expression in wild-type cells. Equivalent experiments were also performed using a monoclonal anti-HA antibody to assess the expression of either of the eIF4Gs in transgenic cell lines, tagged with a C-terminal HA epitope. For the native EIF4G3, a constant pattern of multiple isoforms was clearly seen throughout, with two clear isoforms also seen for the HA-tagged EIF4G3 (Fig. 1A). As for the native EIF4G4, only one clear isoform is observed, although it does seem to increase in intensity after the cells reach the stationary phase. In contrast, for the HA-tagged EIF4G4, a single band was expressed during exponential growth, but a second isoform was clearly seen later, at the stationary phase, starting from 72 h (Fig. 1B). These results are then consistent with both eIF4Gs being possibly targeted by posttranslational modifications which, at least for EIF4G4, may vary according to the *Leishmania* growth phase.

### Phenotypic analysis after knockout of the *EIF4G3* and *EIF4G4* genes

**Fig. 2:**
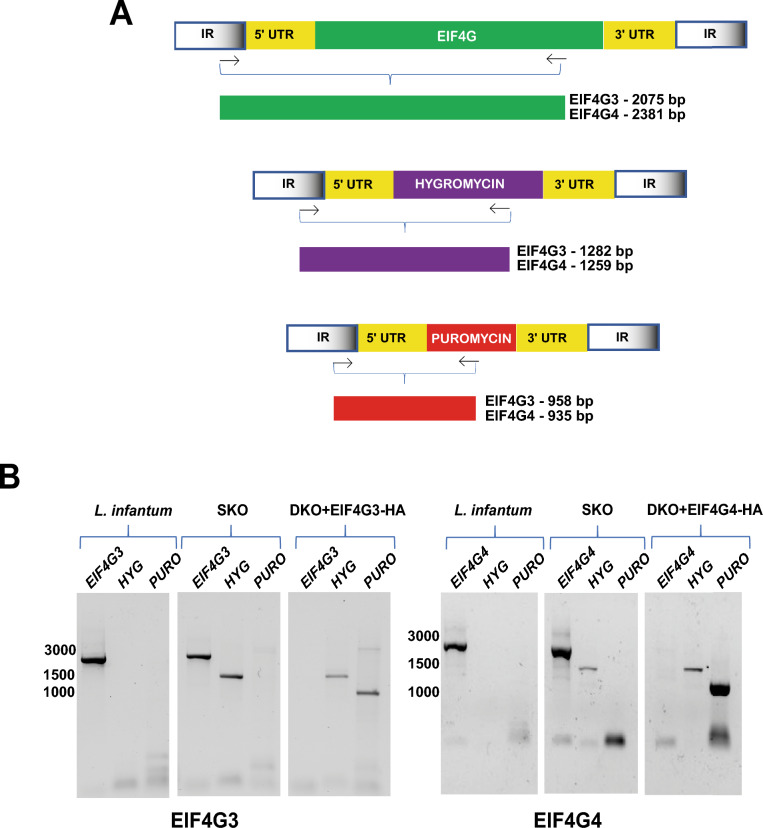
Evaluation of cell lines generated by the knockout and gene complementation experiments confirming the essentiality of *Leishmania* EIF4G3 and EIF4G4. **A** Schematic representation of the endogenous *EIF4G3* and *EIF4G4* genes as well as the corresponding integration events with the hygromycin and puromycin resistance markers. Amplified fragments resulting from PCR reactions set out to confirm the presence of the endogenous genes or the integrated resistance markers are also shown. These reactions were set up with forward primers annealing to the intergenic region of the two eIF4G genes, external to the fragment used for integration, and the reverse primers annealing within the coding regions of the endogenous eIF4G genes or the sequences encoding the hygromycin or puromycin markers. **B** Agarose gel electrophoresis showing the products of PCR reactions confirming the deletions of the endogenous genes. Size markers, in base pairs (bp), are shown on the left. *KO* knockout, *SKO* single knockout, *DKO* double knockout, *HYG* hygromycin resistance marker, *PURO* puromycin resistance marker

*T. brucei* EIF4G3 and EIF4G4 were previously shown to be essential for cell viability through RNA interference (RNAi) assays carried out targeting the procyclic lifeforms [21]. Here, to confirm their essentiality in *L. infantum*, attempts were made to generate single-knockout (SKO) or double-knockout (DKO) cell lines for both sets of *EIF4G3* and *EIF4G4* alleles. SKO clones were readily produced after transfection with hygromycin deletion cassettes, leading to replacement of single alleles with the hygromycin resistance marker (illustrated in Fig. 2A), with integration events confirmed through PCR (Fig. 2B). Attempts were then made to generate DKO cell lines by transfecting the SKO cell lines with cassettes having the puromycin resistance gene, but despite three independent attempts to knockout either of the eIF4G homologs, no viable cells were recovered.

Complementation experiments were then performed, with SKO cell lines ectopically expressing the HA-tagged eIF4Gs used for the second gene deletion event. After transfection with the corresponding puromycin deletion cassettes, and for both sets of *EIF4G3* and *EIF4G4* alleles, cell lines with deletion of the two endogenous *loci* were recovered efficiently, with integration of the puromycin and hygromycin markers also confirmed through PCR (Fig. 2B). These results show that the DKO procedures are only possible in the presence of the ectopically expressed tagged proteins, confirming that both eIF4Gs are essential for survival of the *Leishmania* promastigote life stage. Since no relevant growth phenotype was noticed for the complemented DKO cell lines, in comparison with the parental wild-type or SKO cells (Additional File 2: Supplementary Fig. 5), these experiments also validate the functionality of the HA-tagged proteins, replacing the native eIF4Gs without any significant impact on promastigote growth.

### Pull-down assays to investigate proteins associated with the HA-tagged eIF4Gs

We recently carried out proteomic analyses of the EIF4E4/EIF4G3 and EIF4E3/EIF4G4 complexes from *T. brucei*, defining specific proteins coprecipitated with each complex [31]. Some of the identified associations have been validated by multiple approaches, such as those of EIF4E4/EIF4G3 with EIF4AI, PABP1, and RBP23 [21, 30, 33–35, 40]. Others are not as well-defined and might be restricted to *T. brucei* or might be impacted by the methods used. To confirm relevant new interactions for these two complexes, and to identify conserved or divergent features between major trypanosomatid lineages, we next set out to investigate protein partners associated with the HA-tagged EIF4G3 and EIF4G4 from *L. infantum*. To also assess whether the cellular growth phase might impact the identified interactions, we opted to compare complexes from promastigotes recovered at selected time points during culture growth: 24 h (early exponential growth), 48 h (late exponential), and 96 h (stationary phase). Previously, exponential growth in *Leishmania* promastigotes has been found to be associated with a polysome profile indicative of robust translation and which is severely altered, reflecting limited translation, in stationary phase cells [35]. Here, cytoplasmic extracts were first prepared under detergent-free conditions for all three time points from each cell line expressing the tagged proteins, as well as the parental control cells. Extracts were then used to immunoprecipitate the HA-tagged eIF4Gs with anti-HA beads, with coprecipitated proteins identified through mass spectrometry (summarized in Additional File 2: Supplementary Fig. 6). As detailed in “Methods” section, consolidated lists of the coprecipitated proteins were then generated, with the results also evaluated through principal component analysis (PCA). Three replicates were generally assessed for each cell line/time point, with the exception of the 24 h time point for the control samples. For this time point, only two replicates were used, owing to the loss of the third replicate during the experimental procedures, but the PCA analysis showed a profile that corroborates the viability of these samples to be analyzed as valid replicates (Additional File 2: Supplementary Fig. 7).

### Overview of proteins coprecipitating with *L. infantum* EIF4G3

**Fig. 3:**
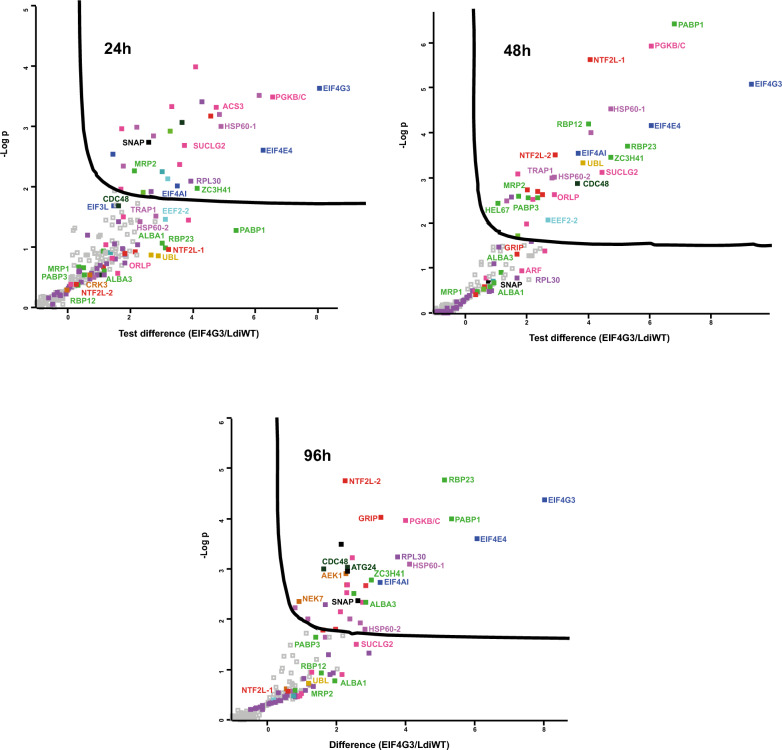
Overview of coprecipitated proteins found with HA-tagged EIF4G3 from *Leishmania infantum* promastigotes. Volcano plot analyses of proteins differentially coprecipitated with the HA-tagged EIF4G3 at different time points. The three volcano plots compare the protein interaction profiles obtained for the EIF4G3-HA bait with the control samples from nontransfected cells. The *x*-axis represents the differences in log_2_-transformed protein intensities between EIF4G3-HA and control, while the *y*-axis shows the −log(*P*) values, reflecting the statistical significance of the observed differences, with the full data set and individual values listed in Additional File 4: Supplementary Table 3. Selected proteins are highlighted, with the colors representing the various functional categories described in the text: translation initiation factors (dark blue); other translation factors (light blue); metabolic enzymes (pink); RNA binding proteins (dark green); uncharacterized proteins (red); ribosomal proteins (dark purple); heat shock proteins and chaperones (light purple); protein kinases (dark brown); proteasome related proteins (light brown); other unrelated proteins (black)

We next set out to define potential protein partners that were most likely to be specifically associated with EIF4G3-HA through immunoprecipitation experiments with anti-HA beads. We therefore compared coprecipitated proteins from lysates prepared from cells expressing the HA-tagged EIF4G3 with those from lysates derived from the control parental cell line. Differences in log_2_-transformed protein intensities between EIF4G3-HA and the corresponding control, with the respective −log(*P*) values, were then calculated for the identified proteins. The data were used to build the volcano plots shown in Fig. 3, for the different time points, with detailed individual values listed in Additional File 4: Supplementary Table 3. EIF4E4, PABP1, EIF4AI, and RBP23 were consistently found coprecipitating with EIF4G3-HA at all three time points, generally being among those proteins with the greatest differences in intensities between the tagged bait and the control and with the most significant −log(*P*) values. Other proteins consistently associated with EIF4G3-HA in all three time points include: one other RNA binding protein, the zinc-finger protein named ZC3H41; several metabolic enzymes, such as phosphoglycerate kinase (PGKB/C) and others; two different heat-shock proteins, homologs of the heat shock protein (HSP) 60 chaperonin, which we arbitrarily named here HSP60-1 (LINF_300033500) and HSP60-2 (LINF_320024300); and other unrelated proteins, such as the vesicular transport protein CDC48 and a soluble NSF attachment protein (SNAP). Several proteins seemed to be specifically associated with the tagged bait only at selected time points. Relevant examples include proteins found with the bait in both time points from exponential growth (24 h and 48 h), such as: the elongation factor EEF2-2; the homolog for the tumor necrosis factor receptor-associated protein 1 (TRAP1) chaperone, or HSP84; a ubiquitin-like protein (UBL); and an uncharacterized protein having a nuclear transport factor 2 (NTF2-like) domain (LINF_180008000), named here NTF2L-1. A second protein having a nuclear transport factor 2 (NTF2-like) domain (LINF_210009700), named here NTF2L-2, was also found associated with the baits, but mostly at the 48 h and 96 h time points. A complex set of likely protein partners, some known and others not previously described, is therefore consistently found with EIF4G3-HA at different time points. Some of those are detailed further in later sections.

### Overview of proteins coprecipitating with *L. infantum* EIF4G4

**Fig. 4:**
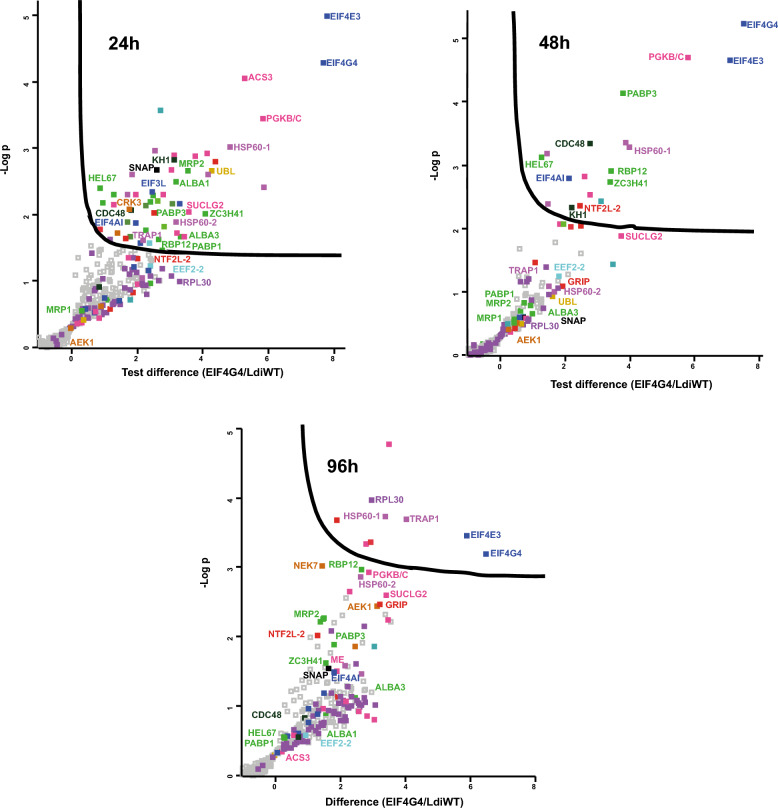
Overview of coprecipitated proteins found with the HA-tagged EIF4G4 from *Leishmania infantum* promastigotes. Volcano plot analyses of proteins differentially coprecipitated with the HA-tagged EIF4G4 at different time points. The volcano plots compare the protein interaction profiles obtained for the EIF4G4-HA bait with the control samples from nontransfected cells, as described for Fig. 3 and with the same color coding. The full dataset and individual values for all represented proteins are listed in Additional File 5: Supplementary Table 4

To define proteins preferentially found with HA-tagged EIF4G4, mass spectrometry data from the corresponding immunoprecipitation experiments were also used for similar analyses as described for the EIF4G3-HA samples. Volcano plots were therefore also built to investigate the proteins coprecipitated with the tagged EIF4G4 at different time points, as shown in Fig. 4, with data from Additional File 5: Supplementary Table 4. The EIF4G4 bait and its EIF4E3 partner were consistently seen among the topmost proteins in all three time points. However, no additional protein was found only with EIF4G4-HA in all three time points. When compared with the EIF4G3-associated proteins, however, the RNA helicase found with the greatest differences in intensities between the tagged bait and the control was not EIF4AI, but another DEAD-Box RNA helicase implicated in translation, a homolog of the human DDX3 and yeast ded1p, alternatively named HEL67 or Ded1-1 [41, 42]. As for PABP homologs, PABP3, instead of PABP1, was found with greater differences in intensities with EIF4G4-HA. Several other proteins found in all, or nearly all, EIF4G3-HA samples were also found with most EIF4G4-HA time points, such as: the RNA-related ZC3H41; various enzymes, including PGKB/C and SUCLG2; and the chaperones HSP60-1, HSP60-2, and TRAP1. Other coprecipitated proteins with a more growth-phase restricted pattern were also seen in common for both EIF4G3-HA and EIF4G4-HA, such as the fatty acyl CoA synthetase 3 (ACS3) enzyme and the CRK3 kinase, found at the 24 h time point; UBL, found more enriched at 24 h and 48 h for both baits; an uncharacterized protein with a GRIP domain (LINF_110005600), also found with both baits but only at 48 h and 96 h; and two other protein kinases, an AGC family serine/threonine kinase (AEK1) and NEK7, found mainly at the stationary phase (96 h). The results for EIF4G4-HA then not only confirm the stability of its interaction with EIF4E3 throughout the *Leishmania* growth phases, at least in the promastigote stage, but also highlight changes in association with several other proteins, less well known, which may be required for changes in activity and regulatory events.

To validate the mass spectrometry data for both EIF4G3-HA and EIF4G4-HA, independent experiments were also performed where equivalent immunoprecipitated samples were tested through Western blots with a monoclonal anti-HA antibody, as well as with available polyclonal antibodies directed to *Leishmania* EIF4E4, EIF4G3, PABP1, and PABP3 (Additional File 2: Supplementary Fig. 8). The results from these experiments confirm the overall validity of the mass spectrometry analyses and the efficiency of the procedures carried out here.

### Investigating the association between EIF4G3 or EIF4G4 and other translation initiation factors

**Fig. 5:**
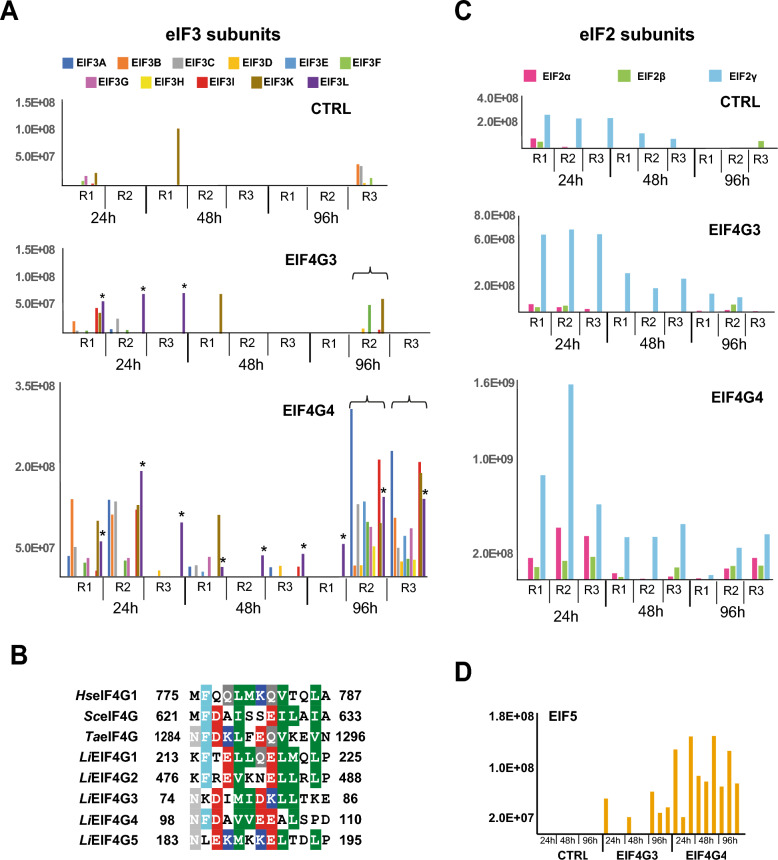
Profiles of selected translation initiation factors coprecipitated with the HA-tagged EIF4G3 and EIF4G4 from *Leishmania infantum*. The graphs plot the normalized intensity values observed for the coprecipitated proteins for the various replicates. **A** Comparison between the various eIF3 subunits coprecipitated with the EIF4G3 and EIF4G4 homologs. The asterisk indicates the profile for the EIF3L subunit, while the braces highlight the replicates from the 96-h time point, for both EIF4G3 and EIF4G4, found to be most associated with ribosomal proteins. **B** Amino acid alignment comparing the defined eIF3L binding motif from the mammalian eIF4G with equivalent sequences found in eIF4G homologs from yeast and wheat, and from all five *Leishmania* eIF4G homologs. Amino acid residues similar or identical in more than 50% of the sequences are shaded with similar colors using the default parameters of the BioEdit software, version 7.0.5.3. Hs—*Homo sapiens*, Sc—*Saccharomyces cerevisae*, Ta—*Triticum aestiva*, Li—*Leishmania infantum*. **C** Coprecipitation profiles for all three eIF2 subunits. **D** Same for the *L. infantum* EIF5. In (A) and (C), the individual replicates are labeled R1, R2, and R3

To better define functionally relevant interactions associated with EIF4G3 or EIF4G4, we first assessed the coprecipitation of the tagged proteins with different translation initiation factors. For this analysis, we only considered coprecipitated proteins with enrichment ratios greater than 1.5-fold with either of the tagged baits and any of the time points investigated, following the criteria described in the “Methods” section (listed in Additional Files 6 and 7: Supplementary Tables 5 and 6, respectively, for the EIF4G3-HA and EIF4G4-HA data). We next searched within these lists for subunits on the eIF3 complex, considering the 12 subunits previously described in trypanosomatids (EIF3A through EIF3L, in capital letters) [43, 44]. With the exception of EIF3J, a loosely associated eIF3 subunit which is not found in any of the samples assessed here, all other 11 subunits are found at various time points. We then opted to directly compare normalized intensity values found for each of these subunits from the three replicates for each growth phase investigated. These were plotted for both EIF4G3-HA and EIF4G4-HA and the corresponding control samples, generating profiles that were easily comparable, as shown in Fig. 5A. Variable profiles were observed for the various subunits, but a greater number of these subunits were found with EIF4G4-HA. This seemed most relevant for the 96 h time point, where two of the replicates (R2 and R3) were consistently found to be enriched with all 11 eIF3 subunits. Overall, EIF3L was the subunit more consistently found for the different time points analyzed and the one generally found with greater intensity values, with its profile highlighted in Fig. 5A. It showed an association with the EIF4G3 at early exponential growth only, while for EIF4G4 it was found in all time points investigated. These results are consistent with a recent report regarding the mammalian eIF3, where the equivalent subunit has been shown to interact directly with eIF4G through a motif found within its MIF4G/HEAT1 domain [45]. The same motif is moderately conserved in the two eIF4G homologs studied here, as seen in the alignment from Fig. 5B, and might indicate a similar interaction for both EIF4G3 and EIF4G4 with EIF3L. Noteworthy also is the moderate conservation of this motif in the remaining *Leishmania* eIF4Gs (EIF4G1, EIF4G2, and EIF4G5).

When other translation initiation factors are considered, in our data, we also saw a consistent enrichment of both tagged baits with the three eIF2 subunits (Fig. 5C), even though EIF2γ was found with intensity values much greater than those observed for EIF2α and EIF2β. All three subunits were also found with greater intensity values with EIF4G4, but for both eIF4Gs they were found with greater values at 24 h, during active cell growth. EIF5 was the only other initiation factor consistently found with the tagged baits and absent from the control samples. It was mainly found with EIF4G3 at 96 h, but coprecipitated with EIF4G4 in all time points assessed (Fig. 5D). Our results are then consistent with the two eIF4Gs having different interaction profiles with relevant translation initiation factors, despite both being active in translation, with these profiles possibly indicating specific functional differences which might also be affected by the *Leishmania* growth phase.

### Assessing the EIF4G3/EIF4G4 coprecipitation with ribosomal proteins and related polypeptides

**Fig. 6:**
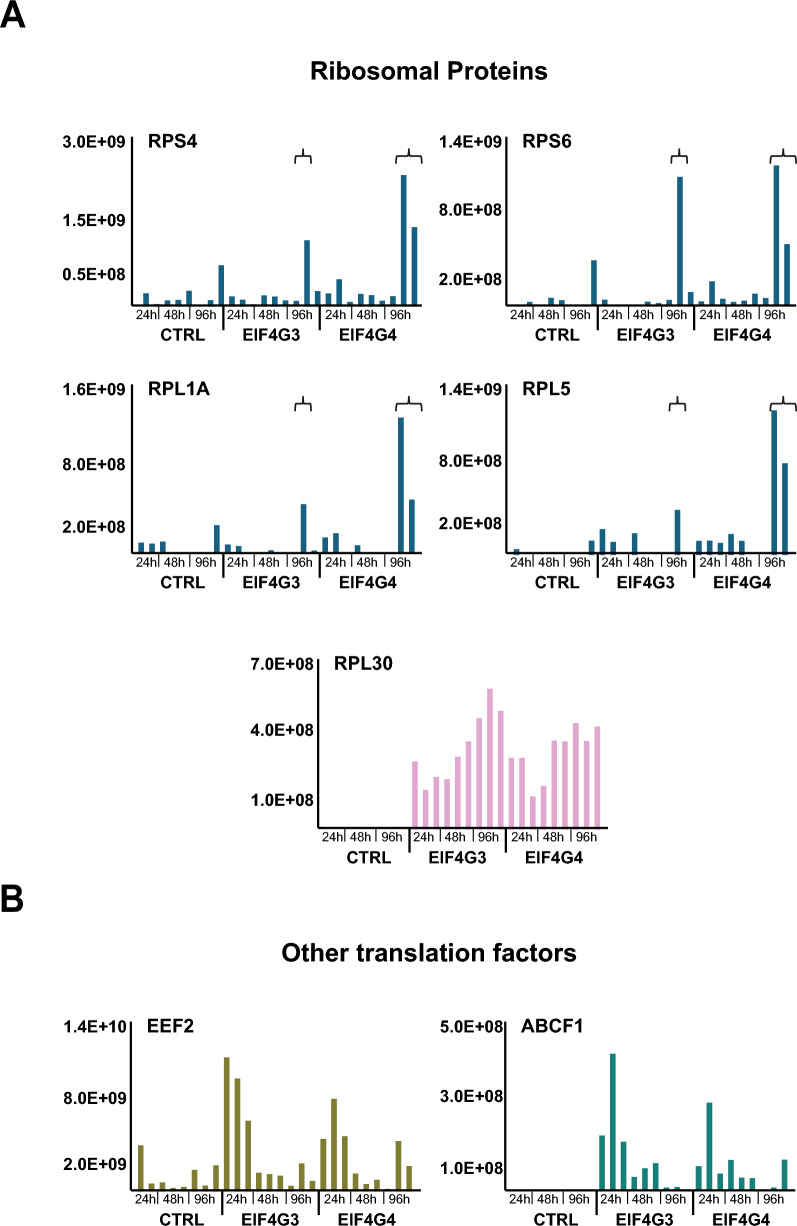
Coprecipitation profiles with the HA-tagged EIF4G3 and EIF4G4 of ribosomal proteins and other translation-related proteins. **A** Comparative analysis of the individual intensity profiles observed for selected ribosomal proteins coprecipitated with the two tagged eIF4G homologs. The braces highlight the replicates from the 96-h time point, for both EIF4G3 and EIF4G4, found to be most associated with the ribosomal proteins, and which are also indicated in Fig. 5A. **B** Individual profiles for the elongation factor EEF2-2 and the translation-related ABCF1 protein

To evaluate whether any similarities or differences in association with ribosomes or polysomes can be defined between EIF4G3 and EIF4G4 during different growth phases, we next attempted to investigate in detail the profile of coprecipitated ribosomal proteins, also among those proteins enriched 1.5-fold or more with the baits. The results showed a loose and variable association for these proteins with the tagged baits, indicating that minor variations in procedure can lead to the baits losing their ability to coprecipitate ribosomes and associated factors. Indeed, most ribosomal proteins were mainly found in one specific replicate for the 96 h samples from EIF4G3 (R2) as well as in the same two 96 h replicates from EIF4G4 (R2 and R3), the ones mostly found associated with the eIF3 subunits. For these samples, more than 40 ribosomal proteins were found coprecipitated with the tagged baits, with individual profiles for selected proteins represented in Fig. 6A and a consolidated profile for all the identified ribosomal proteins shown in Additional File 2: Supplementary Fig. 9. These define what we saw as a “ribosomal” profile, which was useful to identify proteins coprecipitating with the *Leishmania* ribosomes despite a lack of statistical validation. Indeed, this same profile was also seen for several aminoacyl transfer RNA (tRNA) synthetases (Additional File 2: Supplementary Fig. 10). Noteworthy, however, is the exception observed for RPL30, also represented in Fig. 6A and entirely missing from the negative control samples but found with substantial intensity values for all samples with the tagged EIF4G3 and EIF4G4. Other translation-related proteins, such as elongation factor subunits, were also present in the same samples where the ribosomal proteins were found, as expected, and are not considered further here. Nevertheless, some of those were found associated with both baits at distinct time points, such as elongation factor 2 (EEF2-2) and the ATP-binding cassette subfamily F member 1 (ABCF1) protein, both also exemplified in Fig. 6B and found mostly with 24 h or 24 h/48 h time points. Overall, our results not only highlight the loose association between the translation factors and ribosomal proteins for the two tagged baits but also indicate specific proteins that might be involved in more significant interactions, at different time points, which need to be further understood.

### RNA-binding proteins coprecipitated with EIF4G3 and/or EIF4G4

**Fig. 7:**
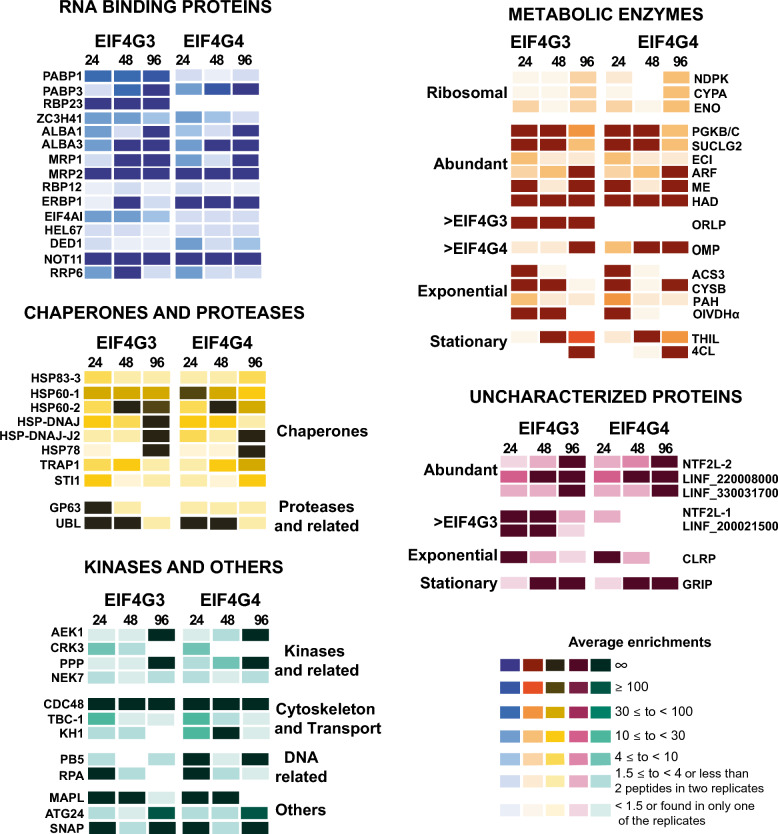
Comparative analysis of selected proteins coprecipitated with the HA-tagged EIF4G3 and EIF4G4. The figure represents proteins from various functional categories, grouped together and represented by different colors, as discussed in the text. For each coprecipitated protein, the average enrichment ratios were calculated based on normalized intensity values obtained for the three replicates from each time point assessed, with the symbol “∞” representing those proteins for which no intensity was defined for the control samples. Individual profiles for all the proteins shown can also be found in Additional File 2: Supplementary Figs. 11–15

We next investigated RNA-binding proteins coprecipitated with the two tagged eIF4Gs and that might be involved in events associated with specific mRNA binding by the corresponding eIF4F-like complexes. For these analyses, and for the ones described in the following sections, we opted to consider more stringent lists of coprecipitated proteins enriched 4-fold or more with EIF4G3-HA or EIF4G4-HA, as described in “Methods” section and listed in Additional Files 8 and 9: Supplementary Tables 7 and 8. We further only considered proteins found in all three replicates and which were statistically validated by the volcano plot analyses (from Additional Files 4 and 5: Supplementary Tables 3 and 4) for at least one of the time points for either of the two baits. Average enrichment values for the most relevant RNA-binding proteins are represented in Fig. 7, with their intensity profiles shown in Additional File 2: Supplementary Fig. 11 and including, for comparative purposes, PABP1, PABP3, and RBP23.

ZC3H41, as previously highlighted, was the RNA-binding protein found with some of the topmost intensities seen for most of the samples, with enrichments generally greater than 4, implicating it as a likely partner for both tagged baits. The two proteins found in *Leishmania *with the acetylation lowers binding affinity (ALBA) domain, ALBA1 and ALBA3, were also found with the two baits in nearly all time points, although ALBA1 was found with higher intensities at 24 h, while ALBA3 seemed to be the most abundant at the stationary phase, despite equivalent enrichments. Also consistently found at most time points with the two baits are, unexpectedly, the mitochondrial RNA-binding proteins MRP1 (also known as the 21-kDa guide RNA-binding protein, or gBP21) and MRP2 (gBP25). Another RBP, also found consistently with the baits, but with lower intensities, is the recently described atypical ERBP1 [46]. The volcano plot analysis uncovered yet another RNA-binding protein, RBP12, also found with the control samples and a low overall enrichment but with significant statistical support for an association with the tagged baits (Figs. 3 and 4, Additional File 2: Supplementary Fig. 11, Additional Files 4 and 5: Supplementary Tables 3 and 4).

We also investigated RNA helicases aside from the already mentioned EIF4AI and HEL67/Ded1-1, but only one is found with any possible relevance, with much lower intensity values, DED1 (also named as Ded1-2—[41]), preferentially found with EIF4G4 (included in Fig. 7 and in Additional File 2: Supplementary Fig. 11). Proteins involved with mRNA deadenylation and degradation, which also coprecipitate with the baits, include the NOT11 subunit of the NOT complex and a possible exosome component (RRP6). Overall, a mixed profile of RNA-binding proteins is then seen with each tagged bait, with several restricted to different time points, and although PABP1 and RBP23 can be defined as specific EIF4G3 partners, no single RNA-binding protein could clearly define the EIF4G4-associated complex.

### Investigating metabolic enzymes, with possible moonlighting roles, associated with EIF4G3 and/or EIF4G4

The large number of metabolic enzymes found here to be strongly associated with the tagged EIF4G3 and EIF4G4 led us to investigate their association further, since they might reflect possible moonlighting roles. Indeed, several of those enzymes coprecipitate with specific baits or time points, allowing us to group them further according to different subcategories, all included in the lists from Additional Files 8 and 9: Supplementary Tables 7 and 8. Individual profiles for selected proteins were arbitrarily chosen among those with greater intensity and/or enrichment values. This selection considered mostly proteins with a known or predicted cytoplasmic localization, defined according to their *T. brucei* orthologs, which are then shown in Fig. 7, with their profiles also represented in Additional File 2: Supplementary Fig. 12. The largest subcategory, consisting of more than 40 proteins, includes those enzymes that were specifically found with the EIF4G3-HA and EIF4G4-HA replicates compatible with the previously described “ribosomal” profile. Three of those, with intensity values equivalent to or greater than those seen for most ribosomal proteins, were nucleoside diphosphate kinase b (NDPK), cyclophilin-type peptidyl-prolyl *cis*–*trans* isomerase (CYPA), and enolase (ENO). A second subcategory includes several enzymes found with both tagged baits at most time points and associated with some of the greatest intensity values, with a profile which we named here as “abundant.” The topmost was phosphoglycerate kinase (PGKB/C), found with intensity values equivalent to those found for the PABP homologs, despite being nearly absent from the control samples. Other enzymes found with the same profile, but lower intensities, include succinyl-CoA ligase beta-chain (SUCLG2), 3 -2-trans-enoyl-CoA isomerase (ECI), ADP-ribosylation factor (ARF), malic enzyme (ME), and haloacid dehalogenase-like hydrolase (HAD). A very distinct profile was observed for a predicted oxidoreductase enzyme (ORLP), found with most of the EIF4G3-HA time points, with relevant intensity levels, but entirely absent from the EIF4G4-HA or the negative control samples (profile named “ > EIF4G3”). An example found preferentially with the tagged EIF4G4 (“ > EIF4G4”), and with greater intensities, was the orotidine-5-phosphate decarboxylase/orotate phosphoribosyltransferase (OMP). Noteworthy also were those proteins found with both baits only during exponential growth (“Exponential”) at 24 h or 24 h/48 h, with relevant examples being the cystathionine beta-synthase (CYSB), fatty acyl CoA synthetase 3 (ACS3), phenylalanine-4-hydroxylase (PAH), and 2-oxoisovalerate dehydrogenase alpha subunit (OIVDHα). Less typical were proteins found with the two baits mainly at the late exponential or stationary growth phases (“Stationary”), but two relevant examples are the thiamine biosynthesis-like protein (THIL) and the 4-coumarate-CoA ligase-like protein (4CL). The observed profiles reinforce the possibility that at least some of these enzymes might function with yet-undefined roles unrelated to their enzymatic activities, but which could be associated with the function of either of the complexes investigated here.

### Assessing uncharacterized proteins specifically coprecipitated with the tagged eIF4G homologs

Many uncharacterized proteins are also found here with EIF4G3-HA, EIF4G4-HA, or both. These were then grouped according to the same subcategories described above for the metabolic enzymes and included in Additional Files 8 and 9: Supplementary Tables 7 and 8. Only two of those fit into the “ribosomal profile” subcategory (LINF_310029900 and LINF_340022200) and will not be considered further here. A selection of proteins with profiles representing other subcategories is shown in Fig. 7 and also represented in Additional File 2: Supplementary Fig. 13. Several were classified as “abundant,” with examples with greater intensities being LINF_210009700, the second NTF2-like domain-containing protein (NTF2L-2); LINF_220008000; and LINF_330031700. Two other uncharacterized proteins are found consistently with EIF4G3 (“ > EIF4G3”): LINF_180008000, the previously mentioned NTF2L-1, and LINF_200021500, but mainly during early and late exponential growth. In contrast, no protein is clearly found associated with the tagged EIF4G4 only. Uncharacterized proteins found with both baits at selected growth phases include: LINF_340011100 (a leucine-rich repeat protein, CLRP), found during exponential growth; and the already mentioned GRIP domain protein (LINF_110005600), found mainly at 48 h and 96 h. Overall, this analysis reveals several proteins which, through their strong association with either or both baits, may be of functional relevance.

### Chaperones and proteases found preferentially associated with the tagged baits

Several protein chaperones, many also known as heat-shock proteins (HSPs), were consistently found here enriched with both eIF4G subunits, at the different time points (shown in Fig. 7 and also represented in Additional File 2: Supplementary Fig. 14). These include a HSP83 homolog (HSP83-3), found with some of the greatest overall intensities, and the two previously mentioned HSP60 homologs (HSP60-1 and HSP60-2), with HSP60-1 reproducibly found with greater intensity values for all time points investigated. Noteworthy also are two DNAJ homologs, with the first one (HSP-DNAJ) found with greater intensity values at 24 h and 48 h, while the second (HSP-DNAJ2) was seen mostly at 96 h. Relevant observations also include the identification of the HSP78 protein, an ATP-dependent Clp protease subunit found mainly with the tagged EIF4G4, restricted to the stationary phase samples. In contrast, the previously mentioned TRAP1 and the stress-induced protein Sti1 were found with more relevant enrichments at 24 h for both baits, and also 96 h for EIF4G4-HA. Most of these proteins have *T. brucei* orthologs found to have a cytoplasmic localization compatible with our results. Two exceptions, however, are HSP78 and TRAP1, with their *T. brucei* orthologs found in the mitochondria.

Another group of functionally related proteins, which were found to coprecipitate with the tagged baits here, were those related to protein degradation, which include many proteins having a “ribosomal” profile. Others, however, showed different patterns of association with the tagged baits. A relevant example, seen for both baits, was a ubiquitin-like (UBL) protein, having a ubiquitin at its N-terminus, associated with very substantial intensity values, mostly from 24 h and 48 h. Another noteworthy association was the GP63 protease, found with both baits at several time points (UBL and GP63 also included in Fig. 7 and in Additional File 2: Supplementary Fig. 14).

### Kinases and proteins with different functional roles found coprecipitated with the HA-tagged EIF4G3 and EIF4G4

We also took a closer look at coprecipitated proteins with possible roles related to phosphorylation (shown in Fig. 7 and in Additional File 2: Supplementary Fig. 15). Several protein kinases are indeed coprecipitated with the two eIF4Gs but found only at specific time points, such as the previously mentioned CRK3, AEK1, and NEK7. Another related protein, a phosphoprotein phosphatase (PPP), was also found mainly enriched with EIF4G4-HA at the 48 h and 96 h time points. Proteins from other functional categories were also identified of possible functional relevance (selected examples also in Fig. 7 and in Additional File 2: Supplementary Fig. 15). These include proteins related to the cytoskeleton structure and intracellular transport, with relevant examples being the already mentioned CDC48; a Rab-GTPase-TBC domain containing protein (TBC-1), found mainly during exponential growth with both baits; and the flagellum-associated kharon1 (KH1), found mainly with EIF4G4-HA at the 24 h and 48 h time points. Two proteins found with the baits with predicted functions associated with the metabolism of DNA were the PB5 endonuclease, mainly found with EIF4G4, and the 51-kDa subunit of the replication factor A (RPA). Various other proteins could not be functionally grouped but also include some that seem to have relevant associations with the tagged baits, such as the membrane-associated protein named MAPL, the autophagy-related protein 24 (ATG24), and the already mentioned SNAP protein.

### Overview of the most relevant proteins coprecipitating with EIF4G3 and/or EIF4G4

**Fig. 8:**
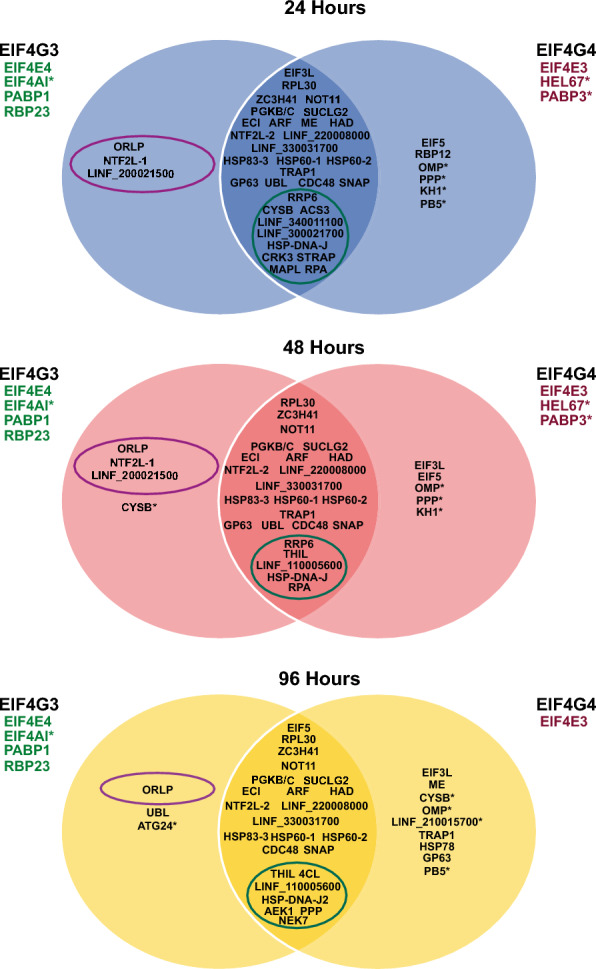
Overview of similarities and differences found for the proteins coprecipitated with the tagged EIF4G3 and EIF4G4 between different *L. infantum* growth phases. The Venn diagrams represent some of the most relevant associations found here for the proteins coprecipitated with EIF4G3-HA and EIF4G4-HA at the early exponential (24 h), late exponential (48 h), and stationary (96 h) phases of the promastigote growth in culture. Omitted are most ribosomal proteins, RBPs, and translation factors, as well as most proteins defined within the “ribosomal” profile. The asterisks indicate proteins found with substantially greater intensity/enrichment values with one of the complexes, but also found with lower values with the second complex, at the same growth phase, despite not  being represented in the figure. The purple ellipses indicate the new proteins found here, specifically associated with the EIF4G3 complex, while the green ellipses indicate those proteins found to have a consistent phase-specific association with both tagged baits

The markedly distinct profiles of coprecipitated proteins found at different stages of *L. infantum* growth curves, observed for both EIF4G3 and EIF4G4, imply significant changes in function/regulation associated with changes in cellular growth. To better define these, we opted to directly compare a selection of proteins most consistently associated here with the tagged baits in the Venn diagram from Fig. 8. The diagram highlights the consistent association between EIF4G3 and its EIF4E4, EIF4AI, PABP1, and RBP23 partners at all three time points assessed, with only EIF4AI also found enriched with EIF4G4, but at lower intensity levels. As for EIF4G4, a consistent association through all three time points is only seen for EIF4E3, although the greater enrichments with HEL67/Ded1-1 and PABP3 during exponential phase time points are also indicated. Other representative proteins are also included in the diagram, such as EIF3L, EIF5, the unusual ribosomal protein RPL30, ZC3H41, and NOT11. Selected enzymes, uncharacterized proteins and others are also represented, such as PGKB/C, LINF_220008000, and the flagellar KH1. The diagram also highlights the three new proteins found in this study to be specifically and strongly associated with EIF4G3: the ORLP enzyme as well as the uncharacterized NTF2L-1 and LINF_200021500. Proteins whose coprecipitation with the baits are most affected by the different growth phases are also indicated, providing an overview of major proteins that might be linked to the regulation of the associated complexes during translation.

## Discussion

So far, and with the possible exception of RBP23, none of the previously identified interacting partners for the EIF4G3/EIF4E4 and EIF4G4/EIF4E3 complexes explain how they identify different mRNA targets, mediate different translation-related events, or might be regulated. Our previous study using *T. brucei* [31] was limited since neither of the eIF4G baits, tagged with the eYFP fluorescent protein, were able to fully replace the endogenous proteins. Both *T. brucei* eIF4Gs might therefore have been functionally impaired. The current study uses baits having the short HA tag, which does not seem to functionally impact the tagged baits, and a different lysis method (cavitation versus grinding). This approach is more reliable and expands on the list of possible interacting partners restricted to each complex or common to both. Many of those partners have so far been overlooked but might assist or regulate either of the investigated complexes in order for them to properly function during translation in *Leishmania* and possibly, but not necessarily, in other trypanosomatid species. For instance, NTF2L-1 and LINF_200021500 were both found previously to be consistently coprecipitated with *Leishmania* RBP23 [30], reflecting their association with the EIF4G3/EIF4E4 complex, but were not investigated further. LINF_200021500 has no identifiable *T. brucei* ortholog, implying functions restricted to *Leishmania* species or that have been lost from *T. brucei*.

The preferential EIF4AI association seen by us with the EIF4G3/EIF4E4 complex has been confirmed through various protein interaction assays carried out with *Leishmania* proteins [20, 21, 23, 33] and has also been confirmed in *T. brucei* [21, 31, 40]. The direct EIF4G4/EIF4AI interaction, investigated for the *Leishmania* proteins, was found to be less efficient, requiring the full-length EIF4G4, while only the MIF4G/HEAT1 domain from EIF4G3 is sufficient to bind EIF4AI [21]. Indeed, the limited EIF4AI coprecipitation seen here with the EIF4G4/EIF4E3 complex was also seen in *T. brucei*, with the preferential association seen here for EIF4G4 with another RNA helicase, HEL67/Ded1-1, as well as the related DED1/Ded1-2, also in agreement with our previous *T. brucei* results [31]. The current data also confirm a greater but not exclusive association for PABP3 with the EIF4G4/EIF4E3 complex. No association could be defined for PABP2, as suggested by previous studies [25, 29, 31], possibly owing to its abundance and/or nonspecific binding to the magnetic beads. It seems likely that the differential association of different helicases and PABP homologs, typical eIF4F partners, to the two eIF4Gs investigated here is related to distinct but undefined functional properties conferred to the corresponding complexes.

Early studies in *Leishmania* have seen EIF4G3, but not EIF4G4, migrating with polysomes [23, 25]. In *T. brucei*, EIF4G3 and EIF4G4 were the only two eIF4G homologs found through a proteomic identification of polysomal proteins, although they were mostly found in the soluble fraction [28], indicating that their association with the mRNAs in polysomes would be transient. Our results are consistent with both eIF4G homologs interacting with the ribosomal subunits during translation initiation, but again through unstable interactions that are not necessarily maintained during immunoprecipitation experiments. A noteworthy observation is the greater degree of association seen here for EIF4G4 with the eIF3 subunits, when compared with EIF4G3, reinforcing its role in translation and in agreement with recent data reported for their *T. cruzi* orthologs [32]. The lack of strong coprecipitation of EIF4G3 with several of those subunits contrasts with previous data where *Leishmania* EIF4G3 was seen to coprecipitate with all 11 eIF3 subunits consistently found for the *Leishmania* complex [44], a difference that might reflect the distinct lysis methods, buffers, and purification procedures. Relevant data regarding the eIF4G/eIF3 interaction in eukaryotes have been described recently through the cryo-electron microscopy (EM) structure of the mammalian 48S complex, when the 40S ribosomal subunit, plus initiation factors, is bound to the mRNA. This structure shows a complex pattern of direct interactions between eIF4G and multiple eIF3 subunits, with eIF3L playing a prominent role, which might be required for the recruitment of the 40S subunit [45]. This contrasts with the data for the yeast eIF4G, which might not bind directly to eIF3 subunits, with ribosomal recruitment proposed to be mediated by indirect interactions involving eIF5, eIF1, and the eIF2β subunit [47–49], and for the divergent eukaryote *Giardia lamblia*, which lacks eIF4G and whose eIF2 complex might help mediate ribosome recruitment [50]. Our results are more consistent with direct interactions between EIF4G3 or EIF4G4 with *Leishmania* EIF3L, resembling the mammalian system and possibly reflecting conservation of the eIF4G/eIF3 interaction in the majority of eukaryotic lineages.

The strong association seen here between ZC3H41 and both tagged baits has not been clearly defined before. ZC3H41 has been found previously to coprecipitate with *Leishmania* PABP1 [35] and has been implicated in translation in *T. brucei* [51], with our results suggesting a functionally relevant contribution to both complexes. The recently described ZC3H41 partner [51], named Z41AP (LINF_050009500 in *L. infantum*), is also found in our analysis at all different time points with both EIF4G3 and EIF4G4, but mostly represented by one peptide in the various replicates, so it was not considered for the subsequent analyses. As for the ALBA domain proteins, they have been previously implicated in translation in both *Leishmania* and *T. brucei* [52, 53], in agreement with their association here with both eIF4Gs. Two other RBPs found here to be associated with the two eIF4Gs investigated here, MRP1 and MRP2, form a dimer that is known to localize within the mitochondria, having been found to associate with mitochondrial mRNAs [54]. MRP1, however, has been found to be part of the mRNA-bound proteome from *T. brucei* and has been classified as a posttranscriptional activator [27, 55], perhaps implying further functions within the cytoplasm which need to be properly defined. On the basis of our results, only a reduced selection of the known RNA-binding proteins found in trypanosomatids are found associated with the two eIF4G homologs, with mostly unclear roles. They potentially act recruiting or defining mRNAs bound by each eIF4F-like complex, but might not be sufficient to allow their different mRNA binding specificities, as previously reported [31, 32]. In contrast, a large number of metabolic enzymes and other proteins found here associated with either or both eIF4G homologs were also identified as part of the extensive mRNA proteome described for *L. mexicana* [56]. Several different metabolic enzymes, including PGK, have also been shown to have moonlighting roles unrelated to their catalytic activities, with several seen to have roles associated with the metabolism of mRNAs and possibly translation [57–59]. Other proteins identified here with possible but undefined RNA-binding activities include the uncharacterized LINF_220008000 and the flagellum targeting protein kharon1 (KH1), both having *T. brucei* orthologs that have been found to be part of the mRNA proteome [27] and were also found associated with polysomes [28]. Although at this stage we cannot rule out nonspecific effects, the coprecipitation with the *Leishmania* EIF4G3 and EIF4G4 of many uncharacterized proteins and others with known functions unrelated to translation, but with possible moonlighting functions, is an indication that at least some of those might have relevant roles related to specific mRNA recognition.

The different isoforms seen for both eIF4G homologs, as well as their differential association with different kinases at different growth phases, imply a possible regulatory role through phosphorylation, consistent with both *Leishmania* EIF4G3 and EIF4G4 being previously identified as phosphoproteins [37]. In *T. brucei*, both EIF4E4 and its PABP1 partner have been shown to be directly regulated by the same cell cycle-regulated kinase, CRK1 [60, 61], and this enzyme has been shown to coprecipitate with both EIF4G3 and EIF4E4 subunits in our previous experiments with *T. brucei* [31]. CRK1 and CRK3 are dissimilar in sequence (47% identity for the *L. infantum* proteins), with both CRK1 and CRK3 previously found to be required for cell proliferation in *Leishmania* and *T. brucei*, and CRK3 found to interact with different cyclin partners (reviewed in Ref. [62]). Since CRK3 is found here to be associated with the two tagged eIF4Gs only during exponential growth, it is unlikely to be directly responsible for the unique EIF4G4 isoform, but it could nevertheless be required to regulate its function. It remains to be seen whether the differences between the CRK kinases found with the two eIF4Gs in *T. brucei* and *Leishmania* reflect true differences that evolved in these parasites. It is noteworthy, however, that a recent systematic study of *Leishmania mexicana* kinases identified CRK3 in the cytoplasm with CRK1 in the mitochondrion [63], supporting the lack of association seen here between CRK1 and the two baits.

This study reinforces the likely functional differences previously implied for the complexes based on the two eIF4Gs investigated here, with the more defined set of binding partners for EIF4G3 in trypanosomatids possibly reflecting its more defined range of mRNA targets. These consist mostly of mRNAs encoding ribosomal proteins, as previously reported [30–32], which are reduced in numbers but mostly very abundant. Binding by the EIF4G3-based complex might allow their translation to be regulated differently than the more diverse set of cellular mRNAs bound by the complex associated with EIF4G4. Overall, the greatly expanded number of proteins identified here associated with either or both eIF4Gs might be relevant for specific aspects of their interactions with mRNAs, ribosomes, and regulators, but it will be important to compare our results with equivalent data for the remaining *Leishmania* eIF4Gs. Indeed, only a selection of proteins, arbitrarily defined to be more relevant, on the basis of translation-related roles or greater association with the baits, were chosen to be individually discussed here. A much larger set of potentially relevant protein partners, however, is found in the mass spectrometry data and might be considered for future research. Our study was limited by the lack of a more detailed investigation on selected potential new partners that were identified here, and their interactions with EIF4G3 or EIF4G4. This will be required to better define true protein partners and their roles assisting specific eIF4Gs in various functional aspects, but this endeavor lies beyond the scope of the present work. The different sets of protein coprecipitated with both baits at different time points may also include novel regulators and reflect shifts in translation activity by the tagged eIF4Gs, which also need to be investigated, but it is important to highlight that some of the changes seen between time points might directly reflect changes in abundance for the coprecipitated proteins. Novel interactions, direct or indirect, suggested by our data, will need to be investigated in more detail, in particular those which might be more functionally relevant. Such studies are also beyond the scope of the current research, but knowledge derived from them can be critical for the design of possible translation inhibitors with potential therapeutic effects, directly targeting *Leishmania* species as well as other pathogenic trypanosomatids.

## Conclusions

Our results confirm the expression of multiple isoforms for EIF4G3 and EIF4G4, with some of those isoforms possibly being regulated during growth, and with both eIF4G homologs being essential for *Leishmania* survival. EIF4G3-HA and EIF4G4-HA coprecipitated with their known eIF4E partners, respectively EIF4E4 and EIF4E3, throughout different growth stages. EIF4G3 more consistently coprecipitated with PABP1, RBP23, and EIF4AI, with EIF4G4 having greater association with PABP3 and the HEL67 helicase. Several likely new EIF4G3 partners were identified but need to be validated, while EIF4G4 seemed to be more strongly associated with other translation initiation factors, including eIF3 subunits. Various potential new regulators of translation, some of those proteins with possible moonlighting functions, were found associated with either or both baits, some only at specific growth phases. Overall, novel functions were implied for previously described as well as uncharacterized proteins, and their characterization might further expand the knowledge on trypanosomatid and eukaryotic mRNA translation.

## Supplementary Information

Additional file 1.

Additional file 2.

Additional file 3.

Additional file 4.

Additional file 5.

Additional file 6.

Additional file 7.

Additional file 8.

Additional file 9.

## Data Availability

The mass spectrometry proteomics data have been deposited to the ProteomeXchange Consortium via the Proteomics Identification Database (PRIDE) partner repository with dataset identifier PXD058985.

## Abbreviations

DKO: Double knockout
eIF: Eukaryotic initiation factor
FDR: False discovery rate
HA: Hemagglutinin
HSP: Heat-shock protein
LFQ: Label-free quantification
PABP: Poly(A) binding protein
RBP: RNA-binding protein
SKO: Single knockout
UTR: Untranslated region
