# Supplementary material for: The related EIF4G3 and EIF4G4 initiation factors from Leishmania: dissimilar modes of action during translation revealed by a comparative proteomic approach
Source: Parasit Vectors. 2026 Mar 4;19:153. doi: 10.1186/s13071-026-07297-1 (PMC13067578; doi:10.1186/s13071-026-07297-1)
Supplement: Supplementary file 2 — Additional file 2. [file 13071_2026_7297_MOESM2_ESM.docx]

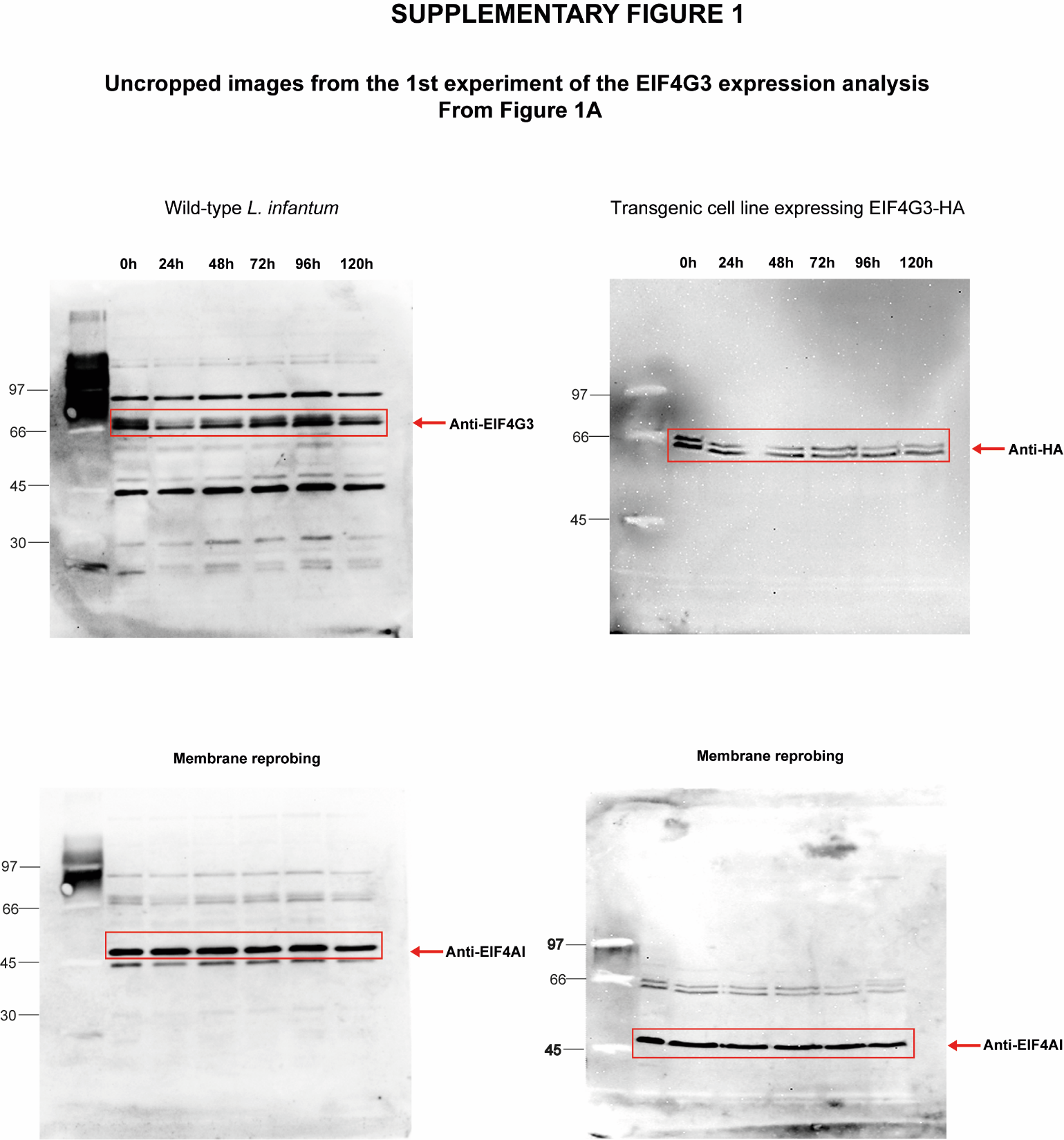


**Uncropped images of the expression analyses of the *Leishmania infantum* EIF4G3.** Detection of the native proteins in wild-type promastigotes was carried out using rabbit polyclonal anti-EIF4G3. The HA-tagged proteins, from the transgenic cell lines, were detected with a monoclonal anti-HA antibody. The native EIF4AI, detected after reprobing with a polyclonal rabbit antiserum, was used as a loading control. The red arrows indicate the relevant bands while the red boxes indicate the cropped segments represented in Figure 1A.


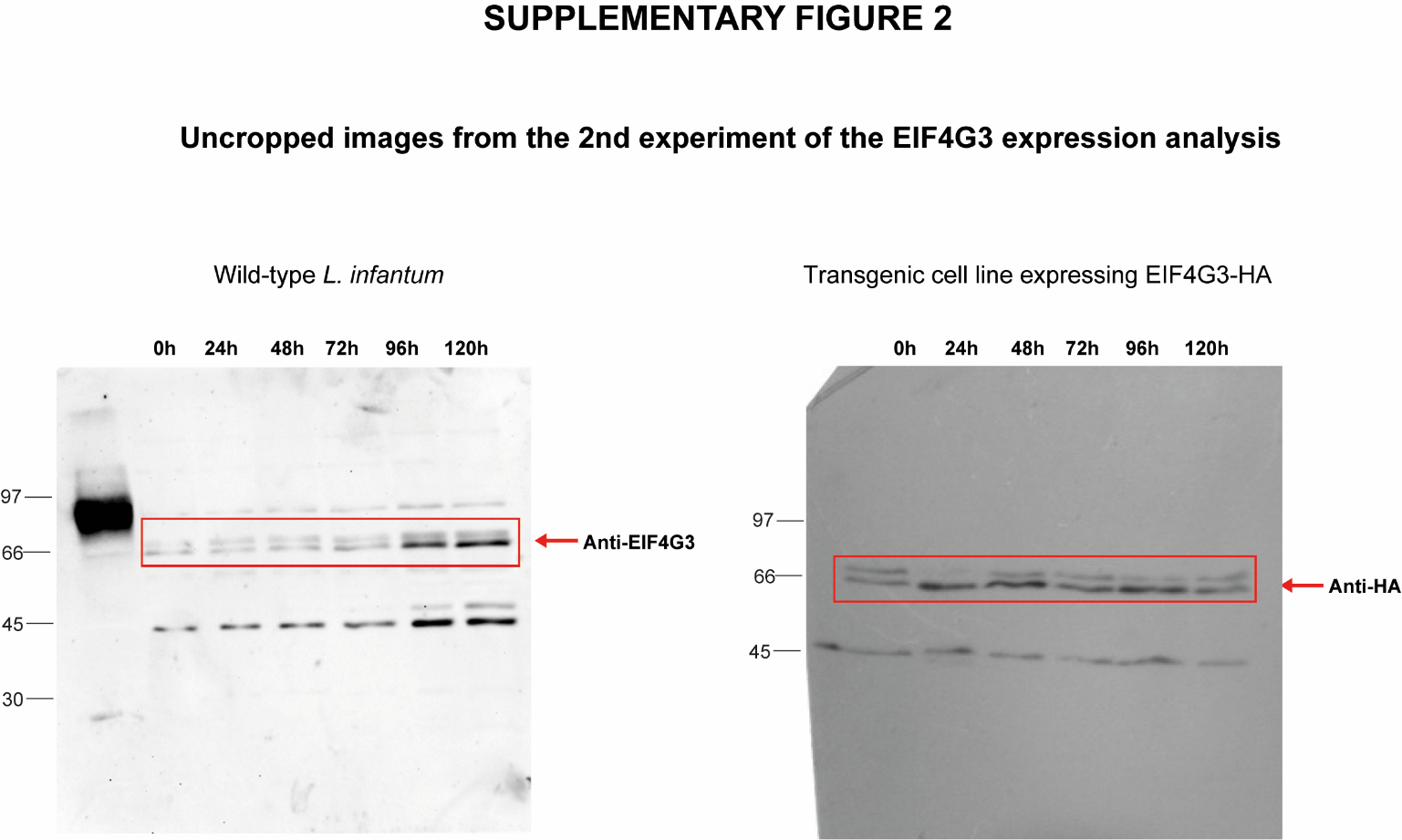


**Uncropped images of a second experiment assessing the expression analyses of the *Leishmania infantum* EIF4G3.** Detection of the native and HA-tagged proteins were performed as described for the Supplementary Figure 1, with the red arrows and boxes indicating the relevant bands.


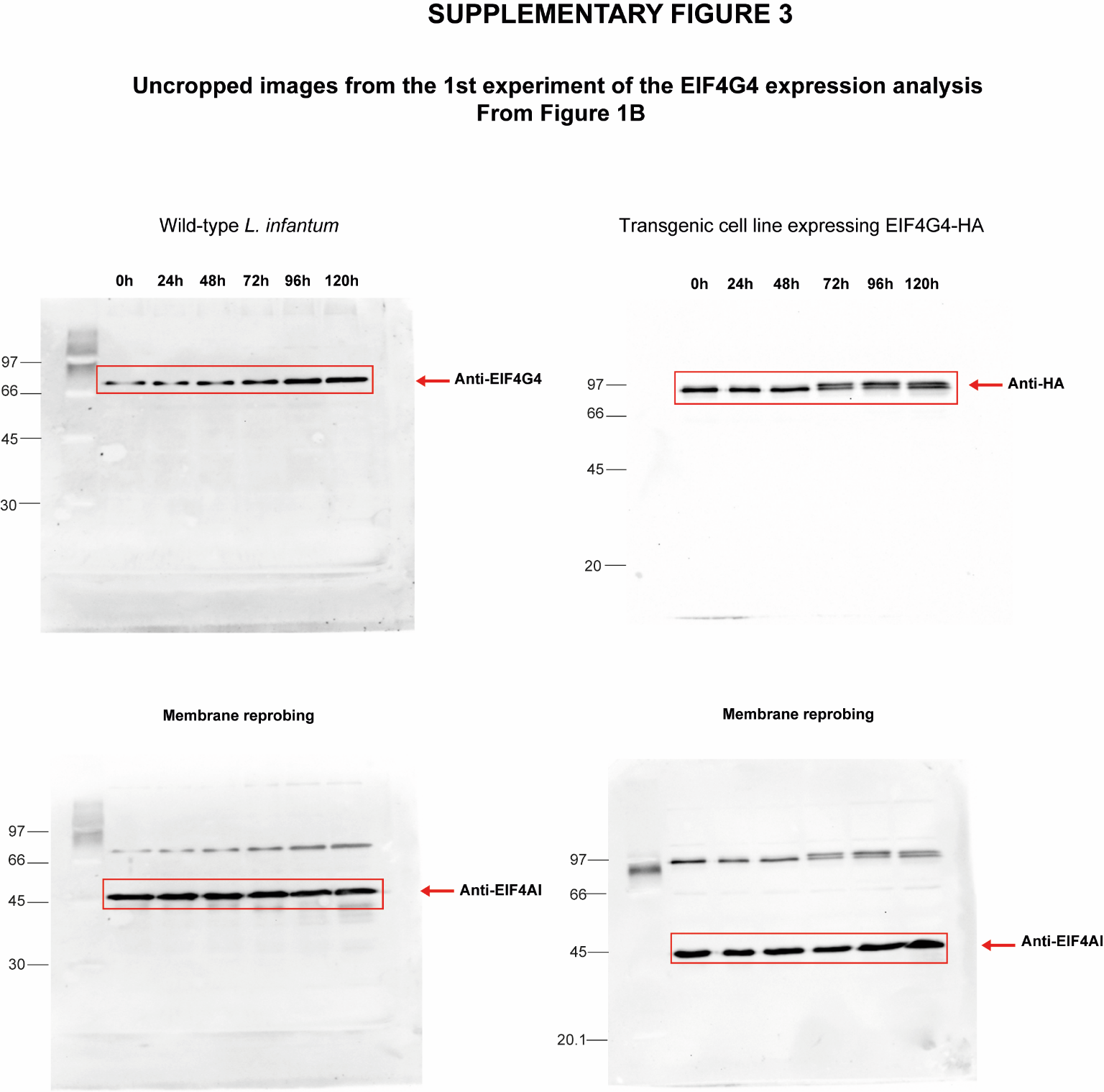


**Uncropped images of the expression analyses of the *Leishmania infantum* EIF4G4.** Detection of the native proteins in wild-type promastigotes was carried out using rabbit polyclonal anti-EIF4G4. The HA-tagged proteins, from the transgenic cell lines, were detected with a monoclonal anti-HA antibody. The native EIF4AI, detected after reprobing with a polyclonal rabbit antiserum, was used as a loading control. The red arrows indicate the relevant bands while the red boxes indicate the cropped segments represented in Figure 1B.


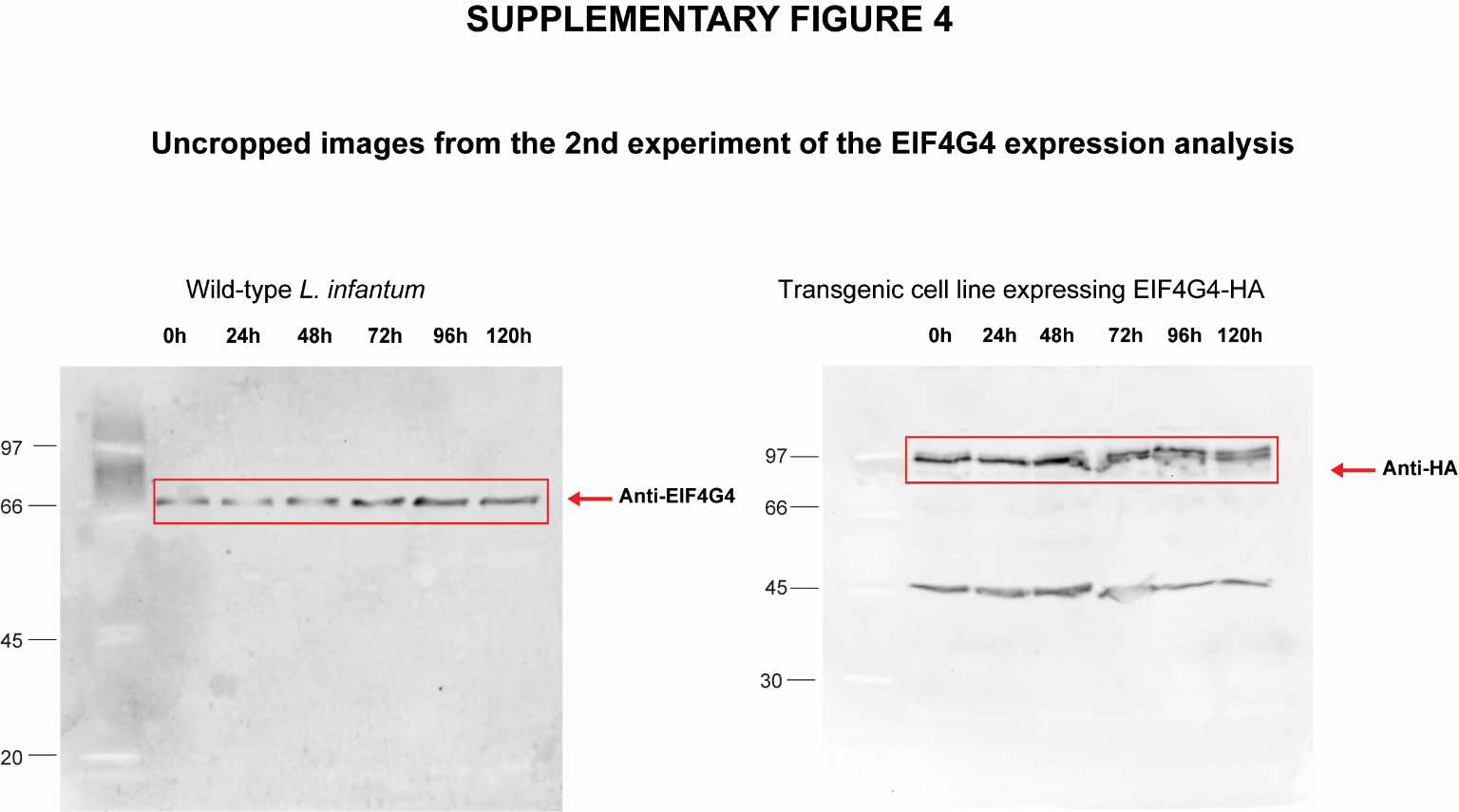


**Uncropped images of a second experiment assessing the expression analyses of the *Leishmania infantum* EIF4G4.** Detection of the native and HA-tagged proteins were performed as described for the Supplementary Figure 3, with the red arrows and boxes indicating the relevant bands.

**
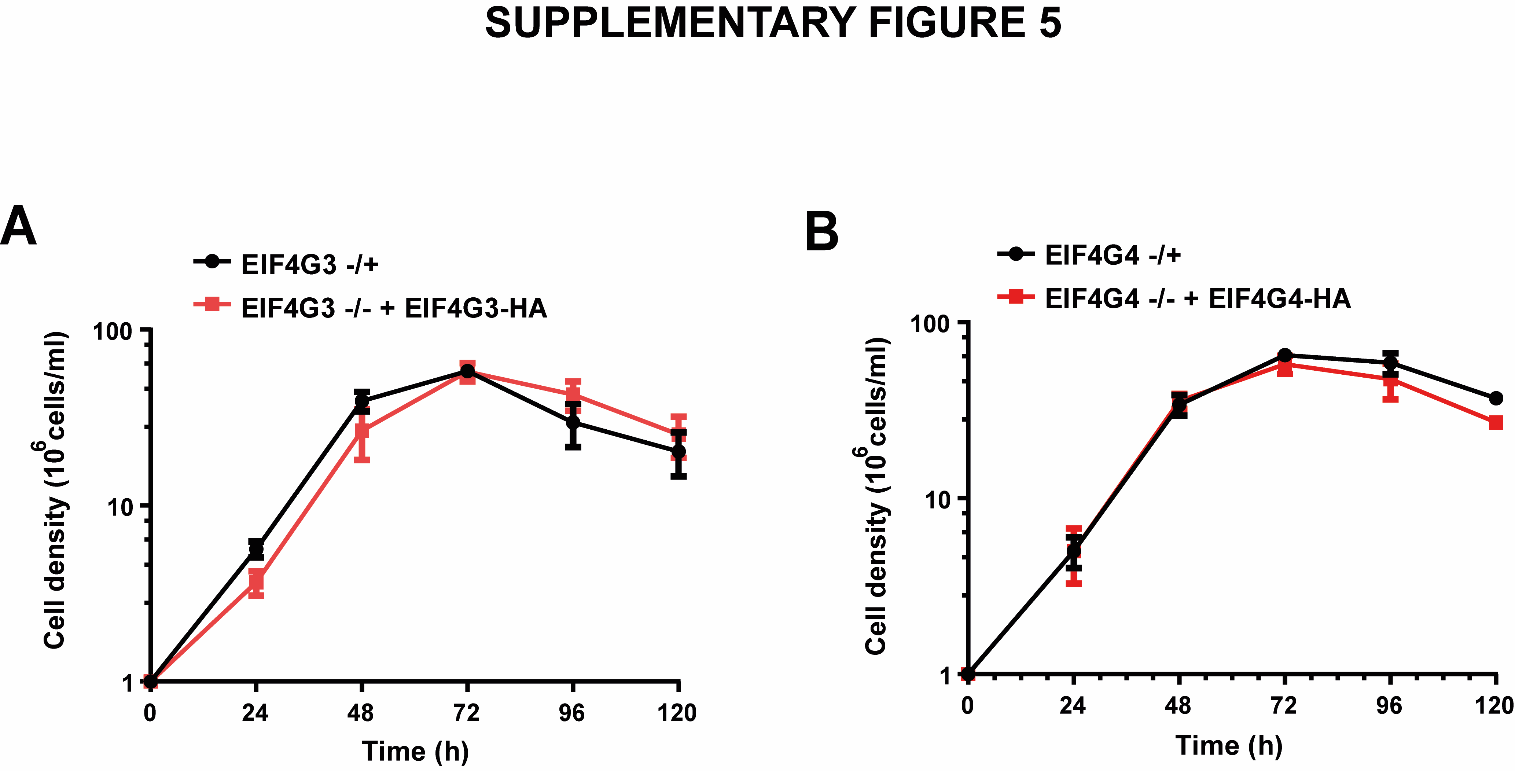
**

**Growth curves of transgenic cell lines of *L. infantum* promastigotes generated after single or double knockout of the *EIF4G3* or *EIF4G4* genes.** The black lines represent the growth curves of the cell lines generated after deletion of single alleles, single knockout (SKO or -/+), of the endogenous *EIF4G3* **(A)** or *EIF4G4* **(B)** genes. The red lines represent the growth curves of the cell lines generated after deletion of both alleles, double knockout (DKO or -/-), of the endogenous *EIF4G3* **(A)** or *EIF4G4* **(B)** genes. The DKO cells lines were only recovered in the presence of the ectopically expressed EIF4G3 or EIF4G4, each C-terminally tagged with an HA epitope (respectively + EIF4G3-HA or + EIF4G4-HA). All growth curves were set up using stationary phase cells diluted into fresh medium to a concentration of 10^6^ cells/ml, followed by monitoring and cell counts at 24 hours intervals.


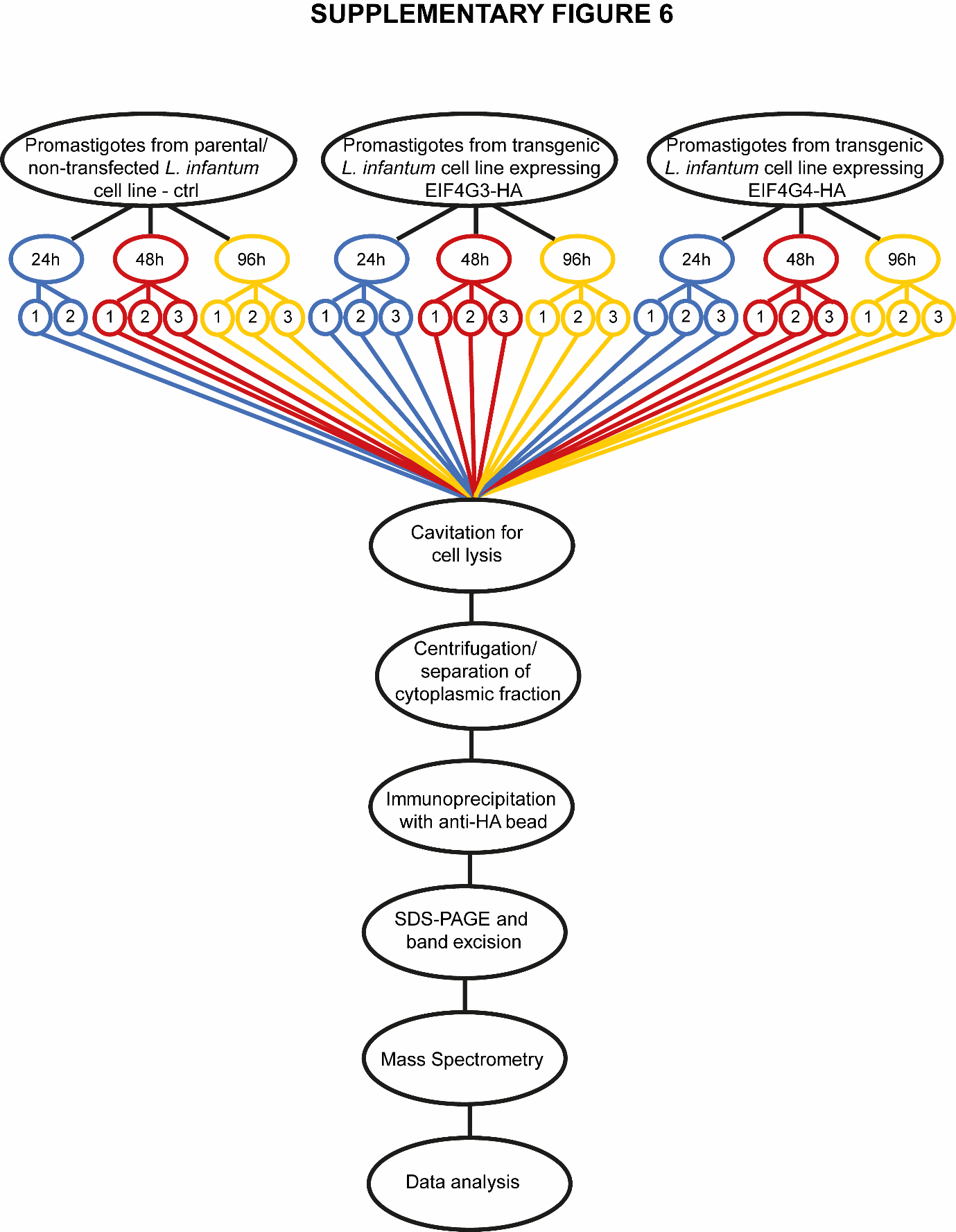


**Organogram representing the major methodological approaches used for the immunoprecipitation/mass-spectrometry analyses.** For each time point, three cultures of *Leishmania infantum* promastigotes were set up for non-transfected/parental controls as well as the transgenic cell lines expressing EIF4G3-HA or EIF4G4-HA (a single culture for the 24h time point for the control cells was excluded during the procedures). At the defined time points three different growth phases were assessed: early exponential (24 h), late exponential (48h) and stationary phase (96h). At these time points the cells were counted and harvested by centrifugation for further processing, as summarized in the figure.


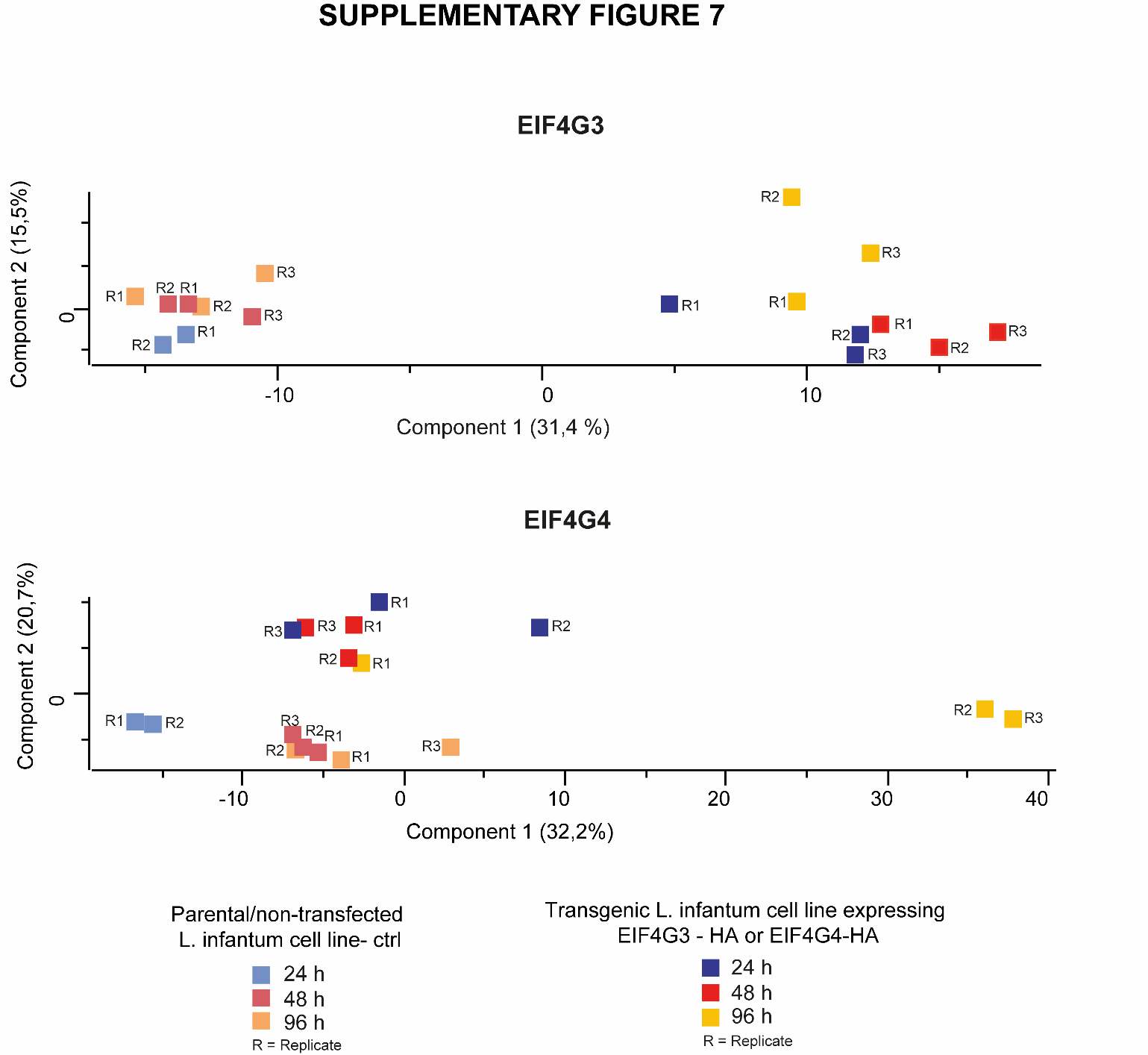


**Principal components analysis (PCA) comparing the mass-proteomic data from the various replicates assayed for the EIF4G3 and EIF4G4 immunoprecipitations.** With the exception of the 24h time point for the parental cell line, with only two replicates, all other time points were represented by three replicates. The distinct profile observed for first replicate (R1) from the EIF4G4 96h time point, when compared with R2 and R3, reflects the lack of co-precipitation with ribosomal proteins, and other proteins, discussed in the text.


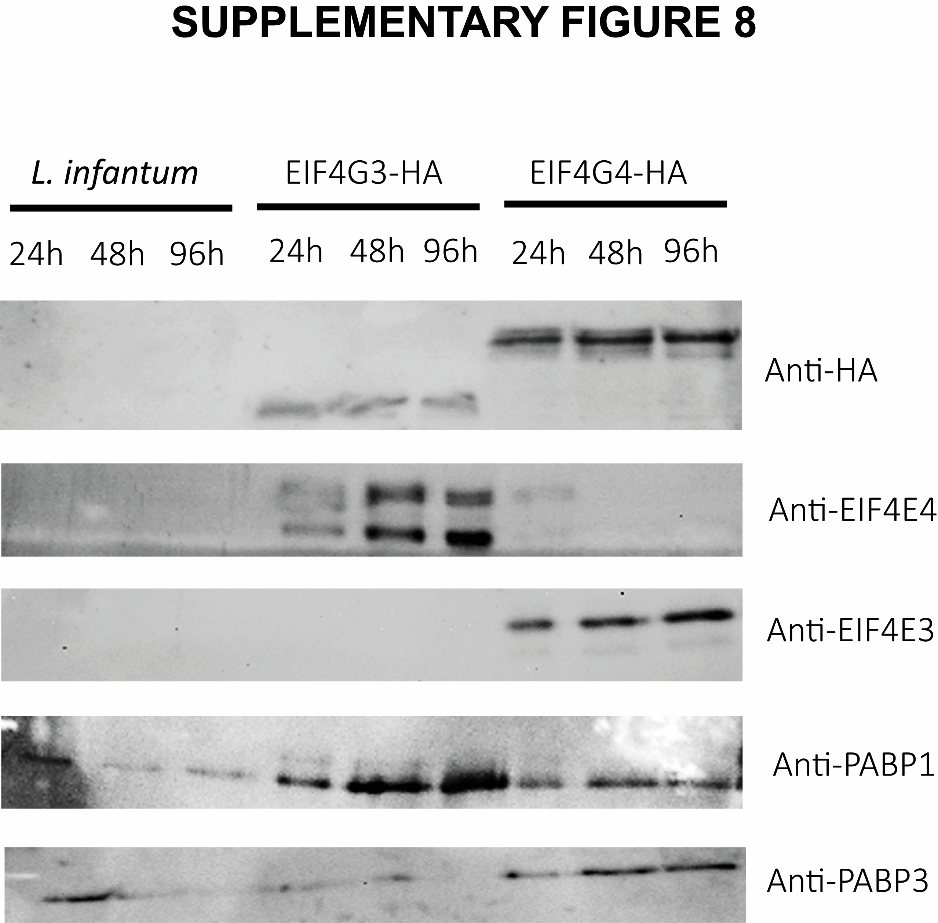


**Western blots confirming the co-precipitation of selected protein partners with the HA-tagged EIF4G3 and EIF4G4 from *Leishmania infantum* promastigotes.** The top panel shows the results of membranes probed with a commercial anti-HA monoclonal antibody assessing the presence of the EIF4G3-HA and EIF4G4-HA in samples of *L. infantum* promastigotes from the corresponding transgenic cell lines, as well as the non-transfected controls, in all three time points assessed (24h, 48h and 96h). The four panels below are from equivalent samples probed with rabbit polyclonal antibodies directed to the *Leishmania* EIF4E4, EIF4E3, PABP1 and PABP3, as indicated. An absence in these samples of clear isoforms, as previously noted for EIF4G3 and EIF4G4, are likely due to the presence of active proteases and/or phosphatases, released during cell lysis and sample preparation, which can lead to some degradation/dephosphorylation of the targeted proteins, and disappearance of isoforms, as also seen for EIF4E3, EIF4E4 and PABP1.


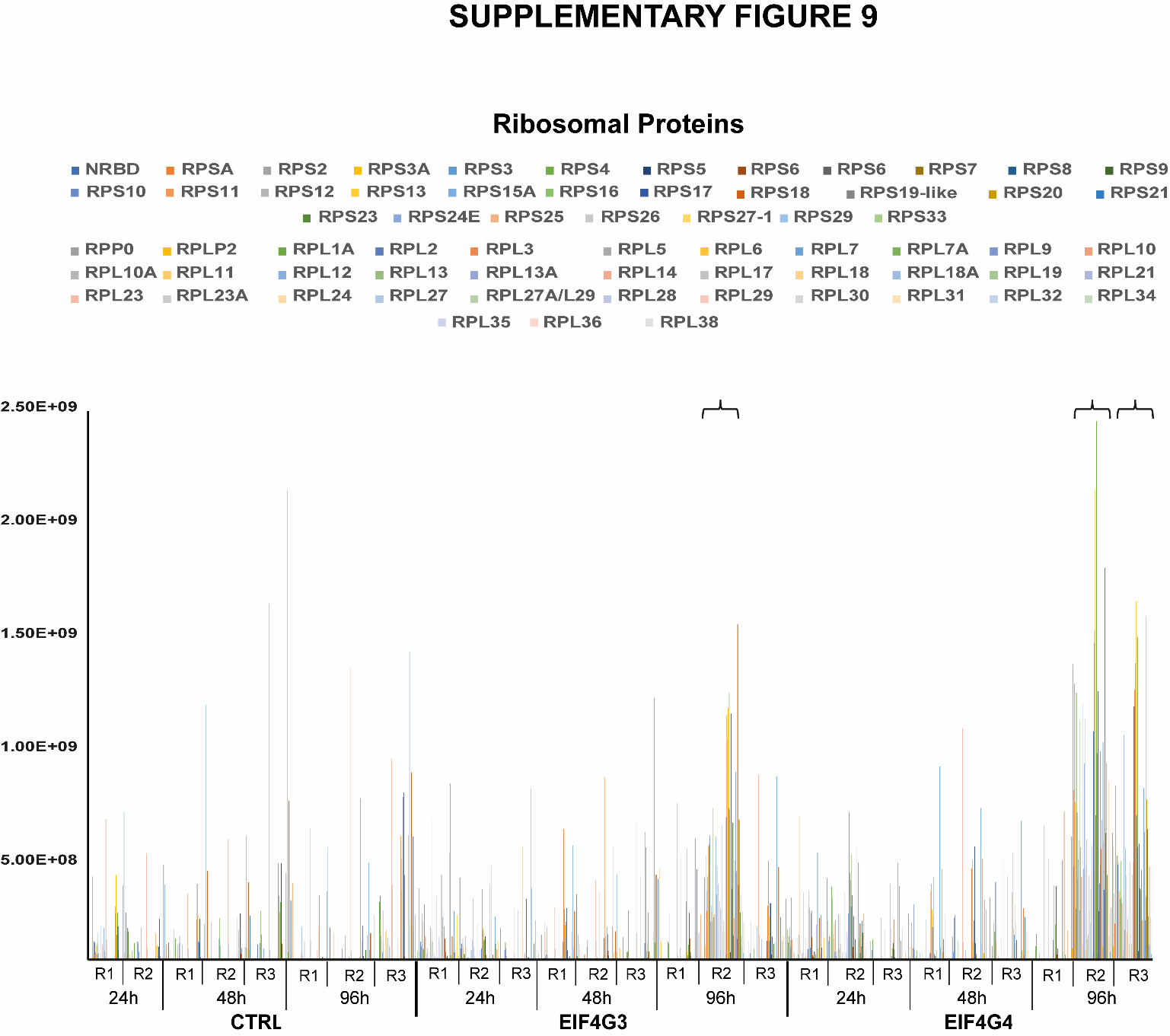


**Profiles of ribosomal proteins co-precipitated with the HA-tagged EIF4G3 and EIF4G4.** The graphs plot the normalized intensity values for the selected ribosomal proteins co-precipitated with the anti-HA beads from each individual replicate for the control cell-line as well as the two transgenic cell lines expressing the tagged eIF4Gs at the replicates from the three different time points assessed (24 h, 48h and 96h). The braces highlight the replicates from the 96h time point, for both EIF4G3 and EIF4G4.


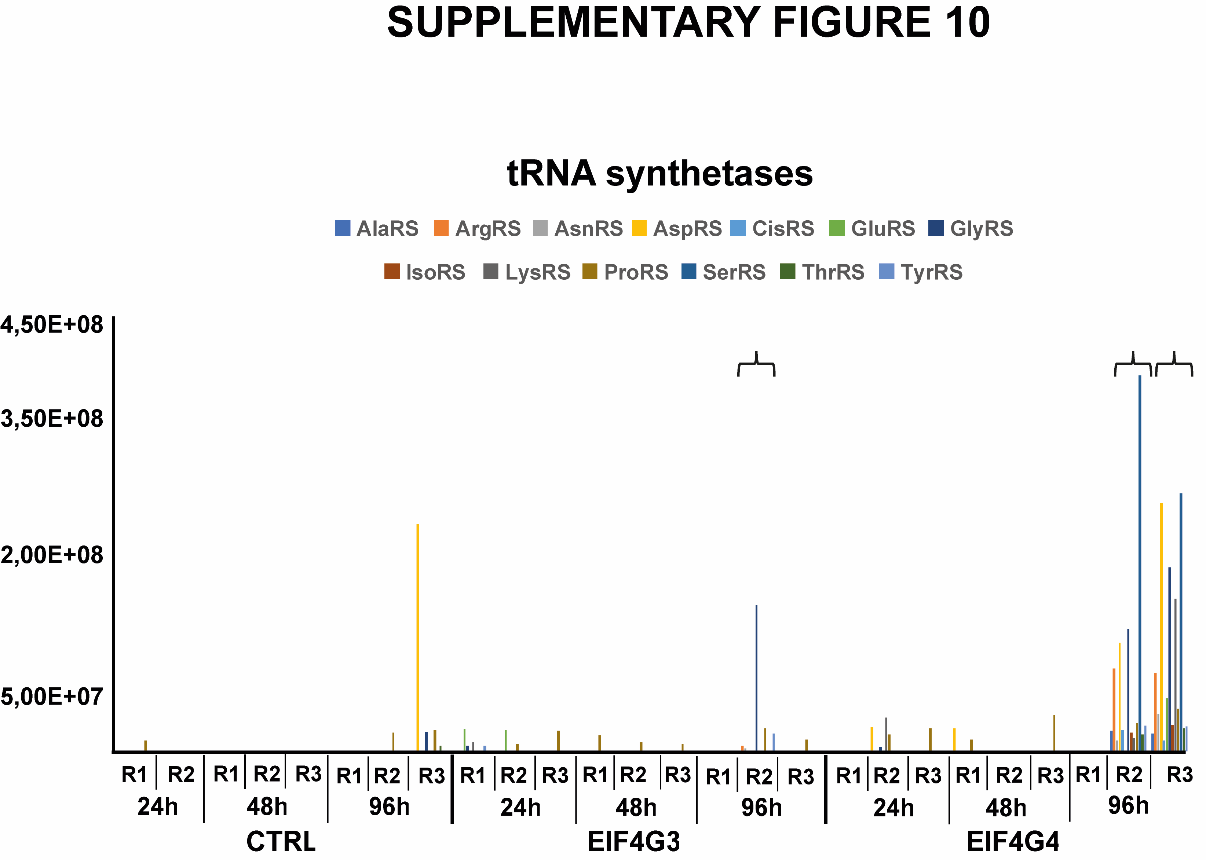


**Profiles of amino-acyl t-RNA synthetases co-precipitated with the HA-tagged EIF4G3 and EIF4G4.** Graphs plotted as described for Supplementary Figure 8. The braces highlight the replicates from the 96h time point, for both EIF4G3 and EIF4G4, found to be most associated with the ribosomal proteins.


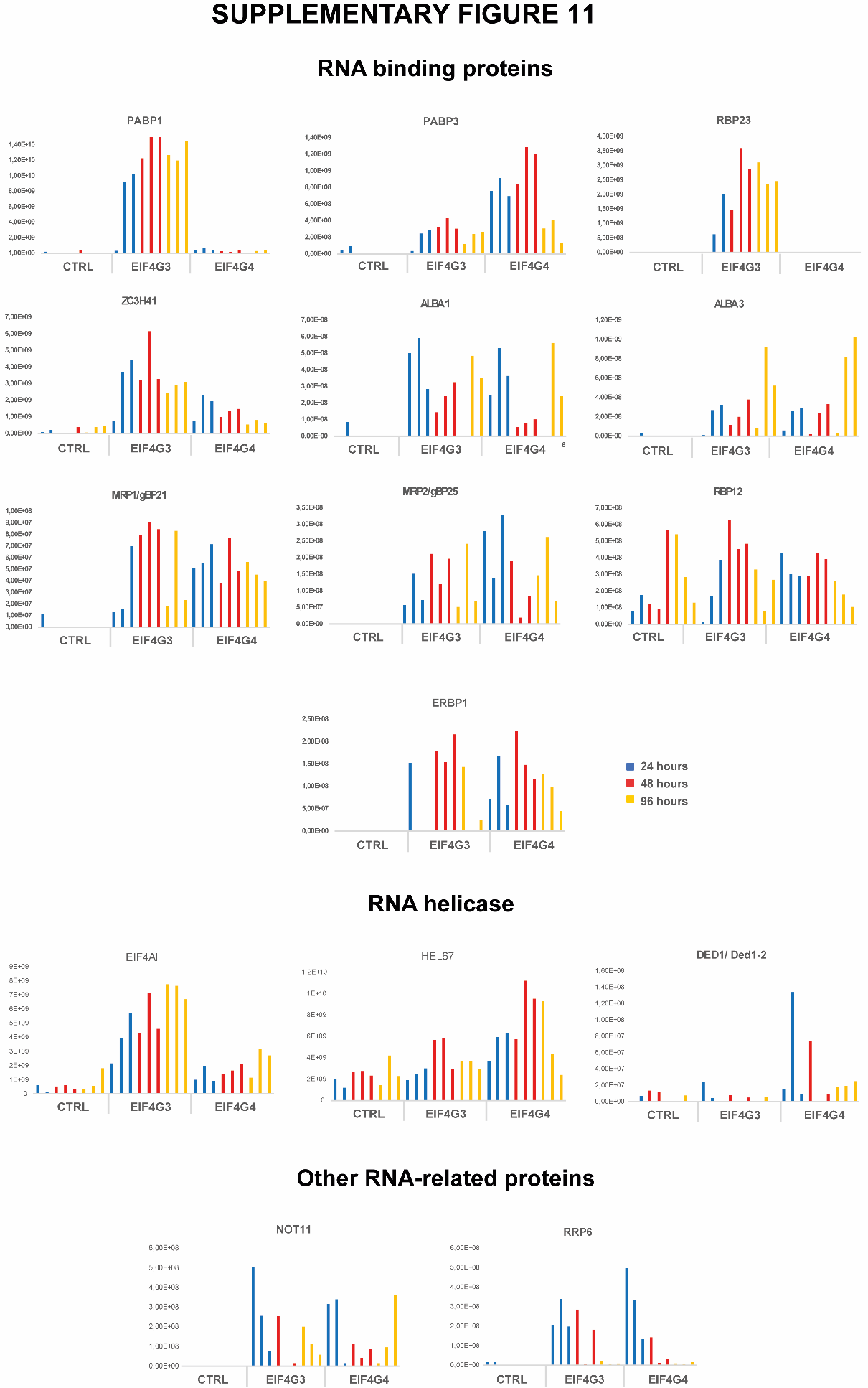


**Selected RNA binding and related proteins co-precipitated with the HA-tagged EIF4G3 and EIF4G4 from different growth phases of *Leishmania infantum* promastigotes.** Graphs plotted as described for Supplementary Figure 8, with the replicates from the three different time points assessed (24 h, 48h and 96h) highlighted in different colors, as indicated in the figure.


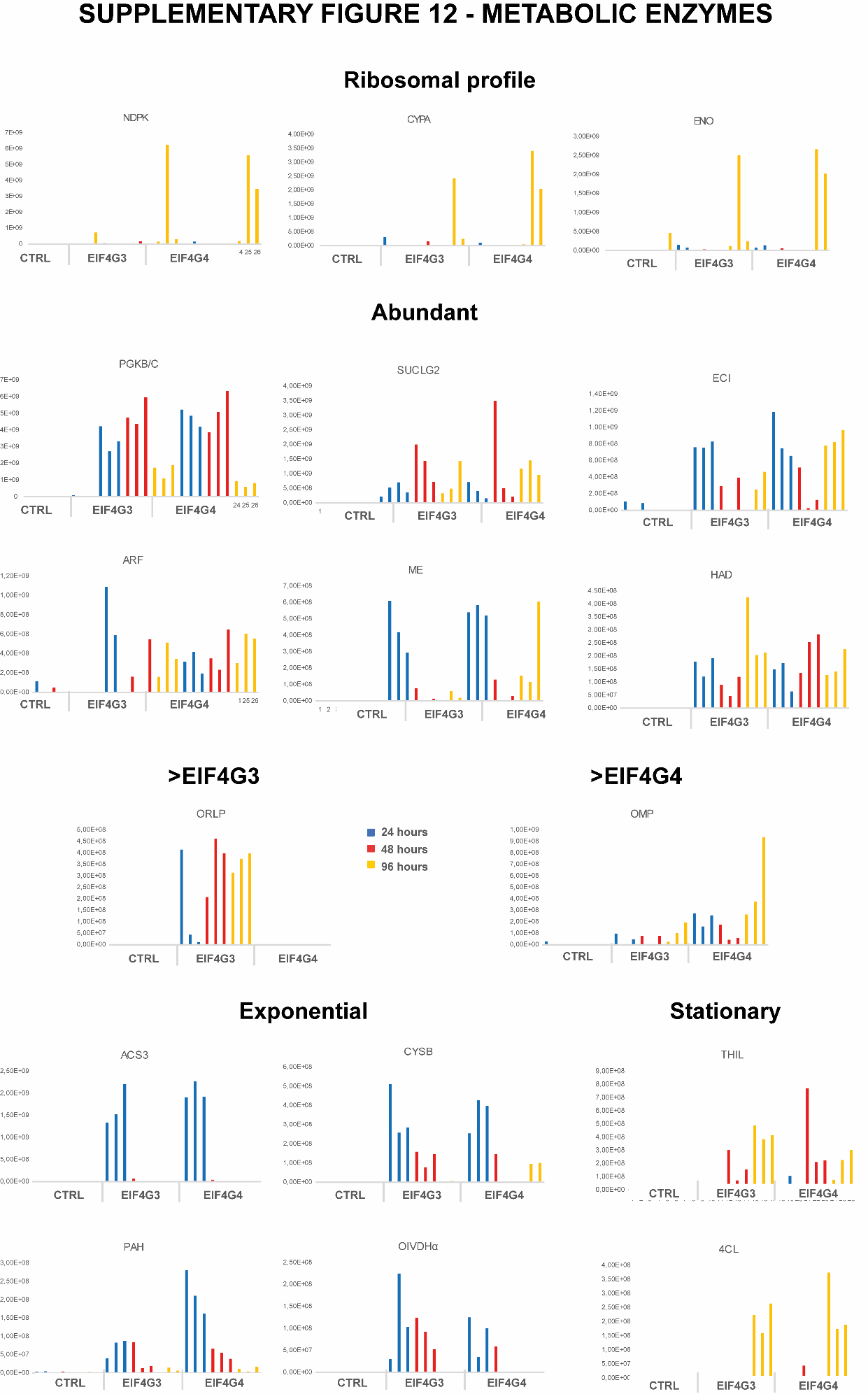


**Selected metabolic enzymes co-precipitated with the HA-tagged EIF4G3 and EIF4G4 from different growth phases of *Leishmania infantum* promastigotes.** Graphs plotted as described for Supplementary Figure 11.


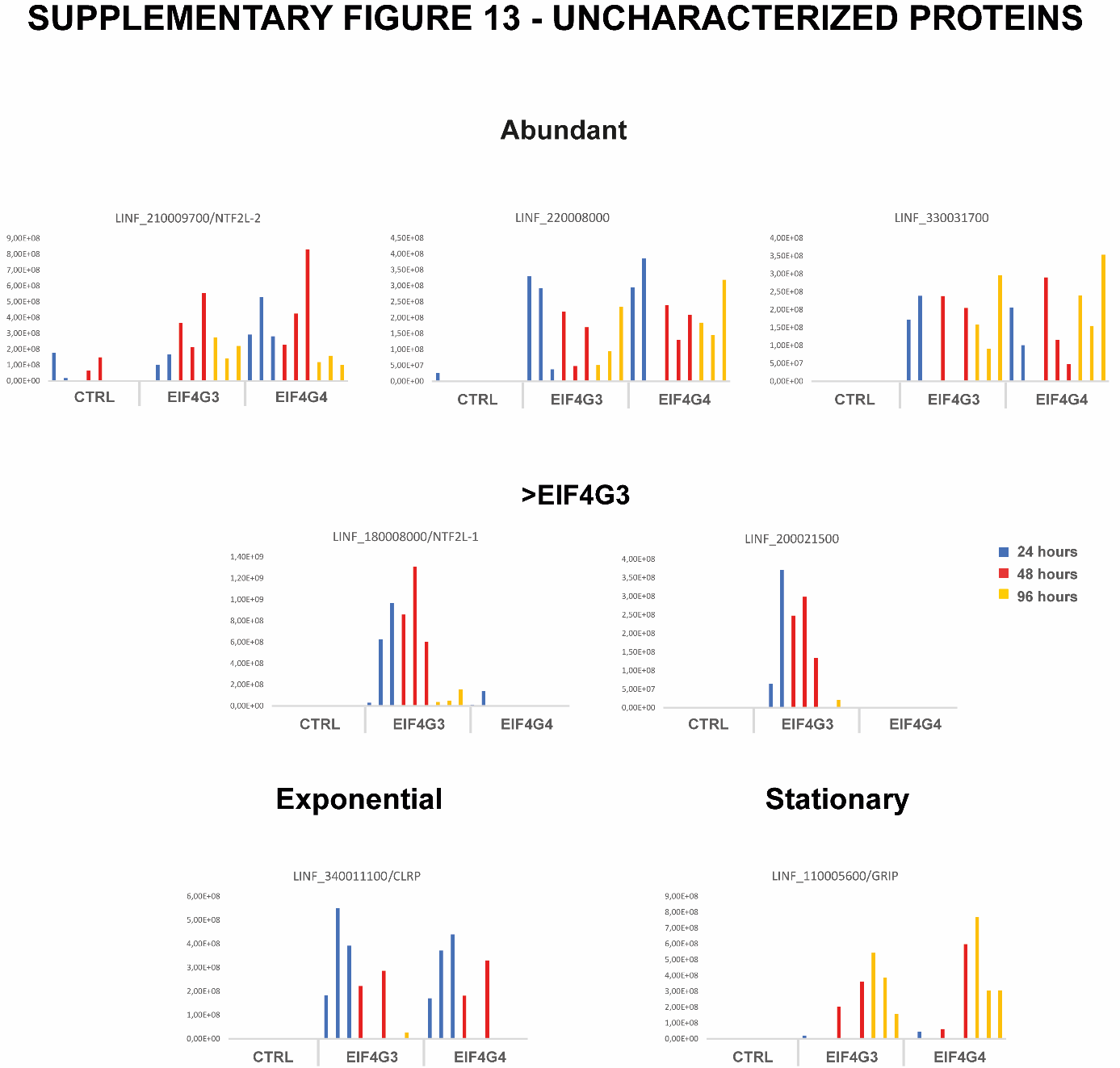


**Selected uncharacterized proteins co-precipitated with the HA-tagged EIF4G3 and EIF4G4 from different growth phases of *Leishmania infantum* promastigotes.** Graphs plotted as described for Supplementary Figure 11.


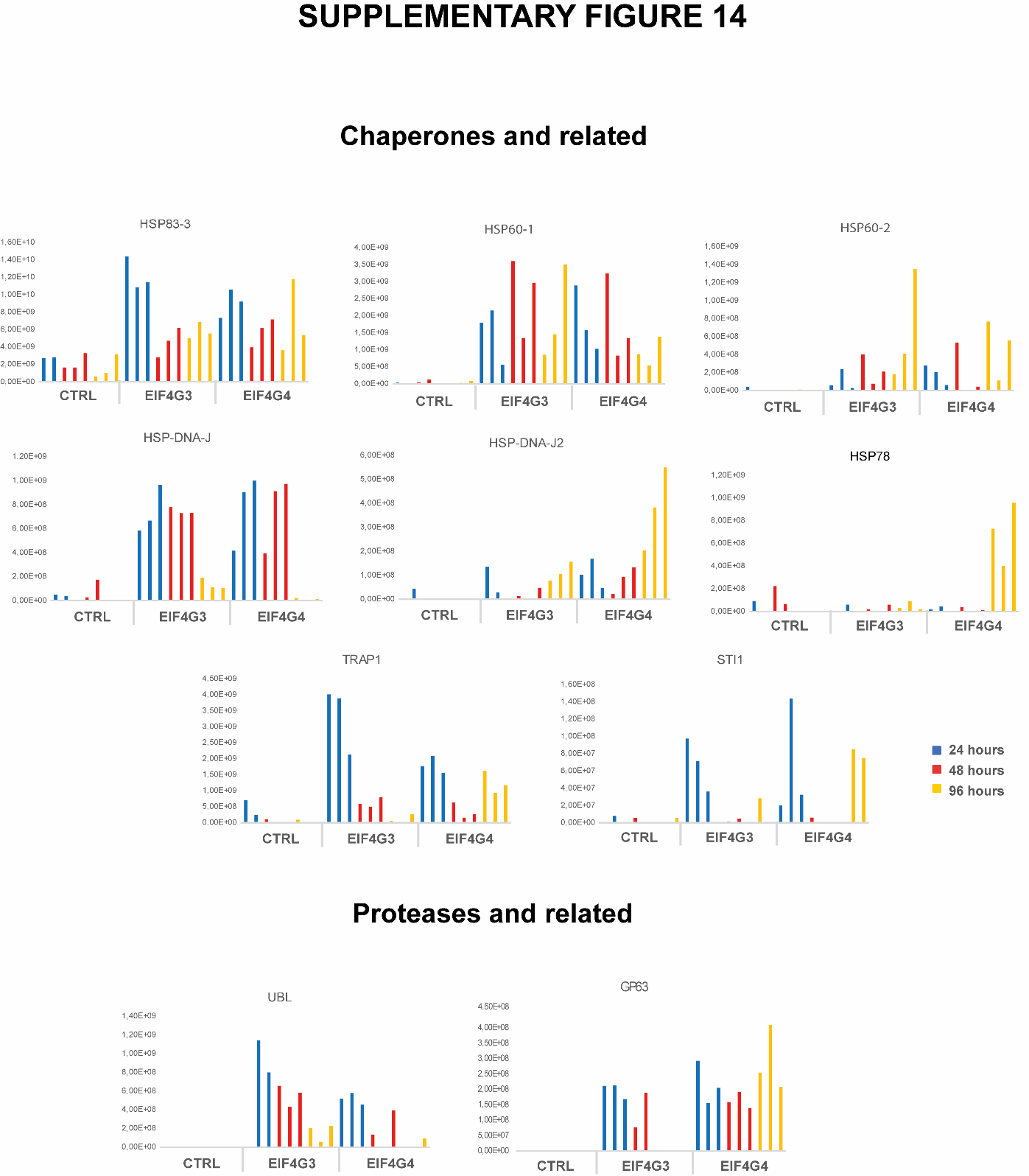


**Selected chaperones, proteases and related proteins co-precipitated with the HA-tagged EIF4G3 and EIF4G4 from different growth phases of *Leishmania infantum* promastigotes.** Graphs plotted as described for Supplementary Figure 11.


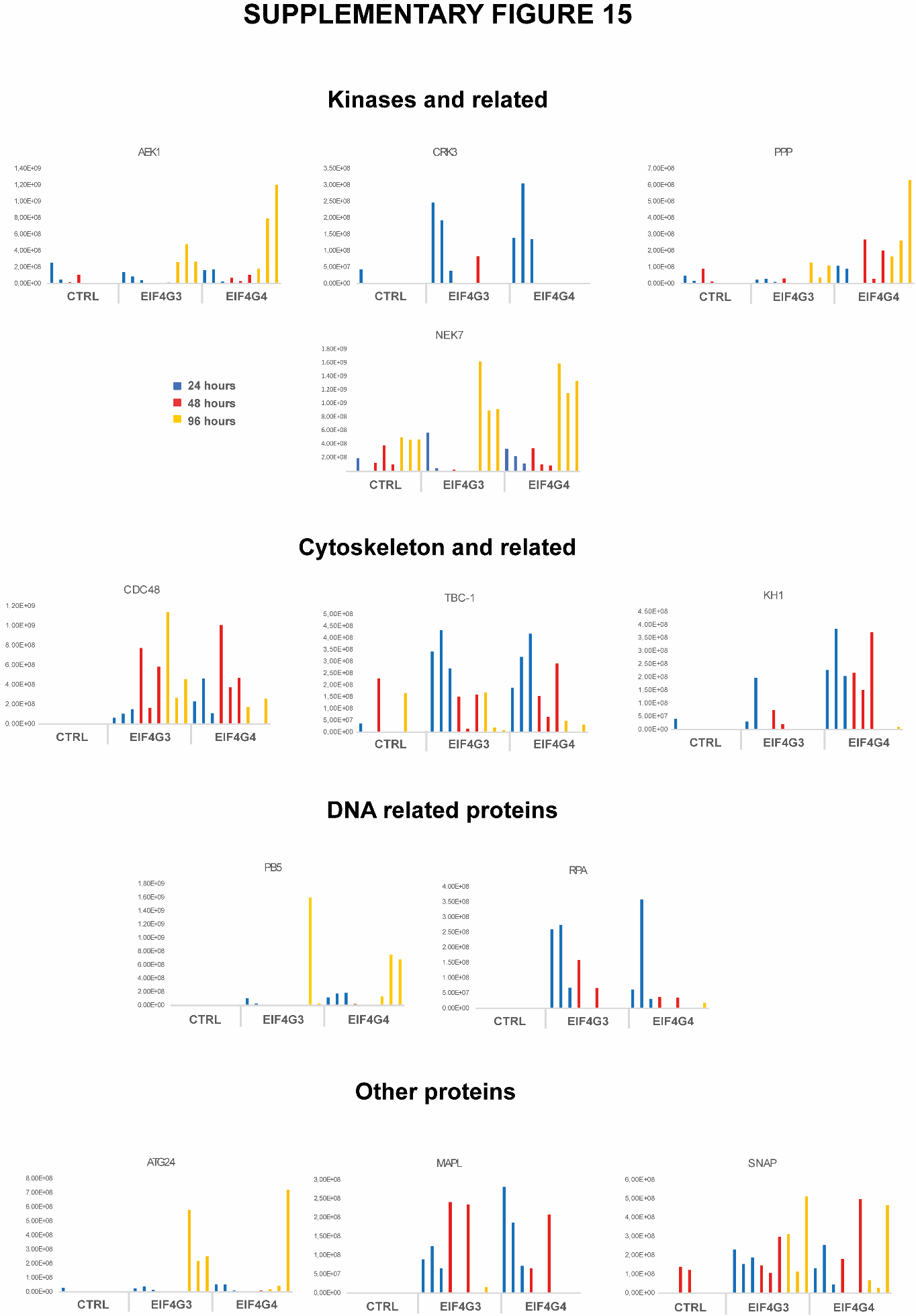


**Selected proteins from various functional categories co-precipitated with the HA-tagged EIF4G3 and EIF4G4 from different growth phases of *Leishmania infantum* promastigotes.** Graphs plotted as described for Supplementary Figure 11.
